# A Frequency-Aware Multi-Stage Framework for Accurate Magnetic Tile Surface Defect Inspection

**DOI:** 10.3390/s26216711

**Published:** 2026-10-23

**Authors:** Zhiyan Zhong, Ye Wang, Jiming Li, Junmin Hu, Hongxin Wang

**Affiliations:** 1 School of Automation and Electrical Engineering, Guangdong Polytechnic Normal University, Guangzhou 510665, China; zhongzhiyan@gpnu.edu.cn (Z.Z.); wangye@gpnu.edu.cn (Y.W.); lijiming02020@stu.gpnu.edu.cn (J.L.); hujunmin8412@163.com (J.H.); 2 Machine Life and Intelligence Research Center, School of Mathematics and Information Science, Guangzhou University, Guangzhou 510006, China

**Keywords:** magnetic tile inspection, frequency-guided gating, local contrast enhancement, co-representation purification, multi-stage framework

## Abstract

Surface defect detection in magnetic tiles is essential for automated quality inspection. However, spatial sparsity of defect regions, pervasive background noise, and low surface contrast lead to weak feature representation, deep feature oversmoothing, and noise amplification under global enhancement. To address these challenges, we propose a three-stage framework integrating localization, enhancement, and segmentation. In the localization stage, we design a Cross-Stage Partial Peripheral Frequency Gating Network (CSP-PFGNet) backbone and an Attention-based Intra-scale Feature Interaction with Wavelet Directional Attention Module (AIFI-WDAM) layer, leveraging frequency-guided gating and directional attention to suppress background clutter and refine multi-scale defect representation. Subsequently, Locally Adaptive Contrast Enhancement (LACE) is applied exclusively within defect regions of interest to amplify contrast while isolating noise, followed by Co-Representation Purification (CoRP) to recover pixel-level defect masks via cross-image commonality mining. Experimental results show that the proposed framework improves ${\mathrm{mAP}}_{50}$ by 1.76 percentage points while reducing parameters and GFLOPs by 11.07% and 11.40%, respectively. In the final segmentation stage, it achieves an IoU of 84.15% and a pixel accuracy of 99.34%, significantly outperforming state-of-the-art baselines.

## 1. Introduction

Magnetic tiles serve as foundational electromagnetic components in permanent magnet motors and servo systems, where their surface integrity directly dictates the operational stability and service life of high-end electromechanical systems [1,2]. Driven by the rapid expansion of new energy vehicles and advanced manufacturing, the industrial demand for high-performance magnetic tiles has scaled into tens of millions annually. However, during harsh processing environments involving high-temperature sintering and precision grinding, magnetic tile surfaces are inherently vulnerable to diverse defects, including cracks, blowholes, breaks, and frays, resulting from raw material impurity fluctuations and mechanical stress [3]. Such localized surface degradation can precipitate catastrophic motor failures and severe operational hazards [4,5,6]. Consequently, developing high-efficiency and high-accuracy automated visual inspection systems for magnetic tile surface defects is of paramount importance to advancing intelligent industrial manufacturing.

**Figure 1:**
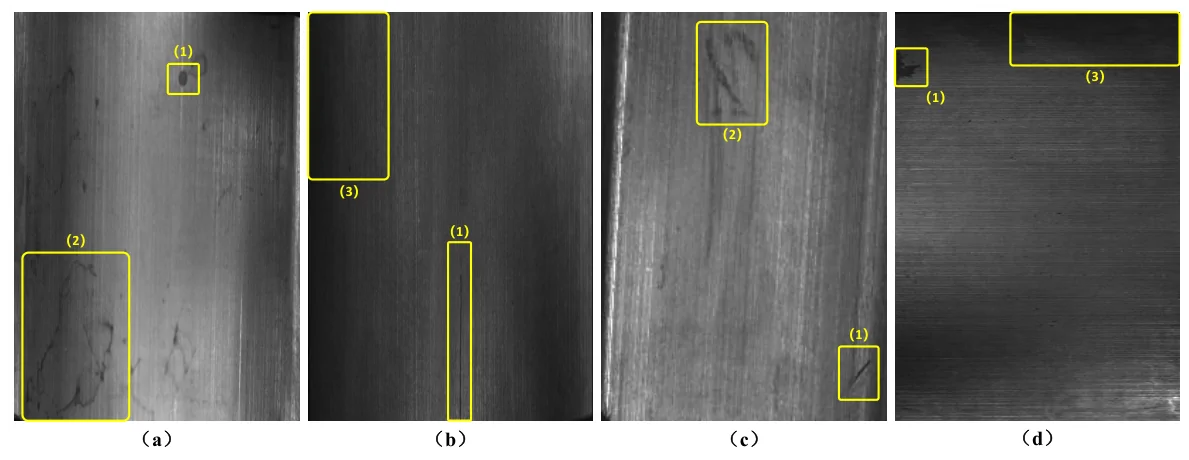
Examples of magnetic tile defects and traits: (**a**) blowhole, (**b**) crack, (**c**) break, (**d**) fray; (1) spatial sparsity of defect regions, (2) complex background clutter, and (3) low surface contrast.

Traditionally, magnetic tile quality control has heavily relied on manual visual inspection by experienced workers under intense illumination [7]. Such human-dependent inspection is inherently subjective, labor-intensive, and prone to both false alarms and missed detections. Consequently, computer vision-based inspection technologies have increasingly supplanted manual routines and are now widely deployed in modern industrial production. Nevertheless, automated detection of the four primary defect categories, namely blowholes, cracks, breaks, and frays, continues to pose formidable technical hurdles. As illustrated in Figure 1, magnetic tile defect imagery naturally exhibits three distinct traits: spatial sparsity of defect regions, complex background clutter, and low surface contrast. These physical attributes collectively give rise to three primary algorithm-level challenges: (1) Weak representation of multi-scale and subtle defects: Small-scale flaws, such as micro blowholes, fine cracks, localized breaks, and slight frays, occupy minimal spatial footprints while displaying substantial morphological variation. Their fragile feature signatures are easily erased during hierarchical downsampling operations, whereas conventional convolutional networks with fixed receptive fields lack the spatial adaptability needed for precise feature capture. (2) Background clutter and feature oversmoothing: Stochastic grinding marks, oil contamination, and uneven surface textures frequently generate false responses that mimic actual defect patterns. Furthermore, standard global feature interaction mechanisms process defect and background features indiscriminately, allowing background interference to overwhelm weak defect signals and leading to severe feature oversmoothing in deep layers. (3) Low contrast and global noise amplification: Defects and ambient surfaces often share overlapping intensity distributions, while non-uniform reflections from curved tiles further suppress defect visibility. Although contrast enhancement can boost defect salience, direct full-image enhancement uniformly amplifies background noise, severely undermining subsequent pixel-level segmentation. Confining contrast enhancement strictly to localized defect regions of interest therefore provides a targeted mechanism to overcome this bottleneck.

In response to the aforementioned core challenges, the primary contributions of this paper are summarized as follows:

(1) We propose a lightweight and effective backbone termed Cross-Stage Partial Peripheral Frequency Gating Network (CSP-PFGNet) to replace ResNet18 in Real-Time Detection Transformer (RT-DETR), enhancing both detection accuracy and efficiency for magnetic tile surface inspection. CSP-PFGNet incorporates a center-suppression filter to mitigate ambient noise alongside a pixel-wise frequency-guided gating mechanism to direct precise spatial attention toward small defect regions. By leveraging multi-scale separable convolutions with expansive receptive fields, CSP-PFGNet captures multi-scale context far more effectively than the baseline ResNet18, substantially strengthening feature extraction within complex background clutter and markedly reducing missed detections and localization errors.
(2) We engineer the Attention-based Intra-scale Feature Interaction with Wavelet Directional Attention Module (AIFI-WDAM) by embedding a Wavelet-based Directional Attention Module (WDAM) into the Attention-based Intra-scale Feature Interaction layer. WDAM seamlessly integrates discrete wavelet decoupling with directional attention, employing high-frequency edge subbands as structural priors to dynamically recalibrate attention maps. This architecture suppresses smooth background textures, concentrates on salient flaw attributes in complex scenes, and mitigates the oversmoothing of subtle defect features.
(3) We establish a unified three-stage framework integrating localization, enhancement, and segmentation, effectively bridging object-level localization and pixel-level mask reconstruction. The customized PFG-RTDETR model accurately crops defect regions of interest (ROIs) for subsequent localized enhancement, spatially isolating extensive background noise and eliminating the noise amplification inherent to full-image processing. Subsequently, Locally Adaptive Contrast Enhancement (LACE) is executed to sharpen local gradient contrast, while the Co-Representation Purification (CoRP) network leverages co-salient object detection to eliminate background clutter, delivering high-precision pixel-level segmentation across complex industrial environments.

The remainder of this paper is organised as follows. Section 2 reviews related work. Section 3 presents the proposed method in detail. Section 4 reports the experimental results on magnetic tile defects, and Section 5 draws conclusions.

## 2. Related Work

Research on detecting small magnetic tile defects under low surface contrast in complex backgrounds can be broadly divided into traditional machine vision methods and deep learning methods. Traditional methods primarily rely on handcrafted features and image processing operators, whereas deep learning methods automatically learn defect representations from data.

### 2.1. Traditional Machine Vision Methods for Magnetic Tile Defect Detection

Early studies primarily employed handcrafted feature operators for background suppression and defect extraction. To enhance weak defects under low contrast and uneven illumination, Li et al. [8] combined the Curvelet transform with texture analysis to suppress grinding textures and detect low-contrast cracks. Xie et al. [9] employed the Shearlet transform and threshold processing to extract defects from complex magnetic tile backgrounds. Yang et al. [10] proposed a stationary wavelet transform method combining Sobel operators with nonlinear enhancement to remove uneven backgrounds. Yang et al. [11] further combined the nonsubsampled Shearlet transform with envelope grey-level gradient filtering for low-contrast cracks. However, these transform-based methods remain sensitive to background textures and parameter settings. To mitigate texture and noise interference, Jiang et al. [12] combined the nonsubsampled Contourlet transform with Laws texture features. Ben Gharsallah and Ben Braiek [13] leveraged anisotropic diffusion to preserve defect boundaries. Furthermore, for small defects in complex backgrounds, Li et al. [14] combined the Contourlet transform with singular value decomposition to strengthen the extraction of small crack features under low illumination. Zhong et al. [15] decomposed images into a low-rank background matrix and a sparse defect matrix using local structural similarity. Zhong et al. [16] further proposed weighted matrix decomposition for small surface defects, constructing a weight matrix from red–green–blue (RGB) channel texture features to better separate defects from the background. Overall, traditional methods advance defect extraction but rely on handcrafted features, fixed processing rules, or structural assumptions, which limits their adaptability to varying defect scales, morphologies, and backgrounds.

### 2.2. Deep Learning Methods for Magnetic Tile Defect Detection

Deep learning has become the predominant approach to magnetic tile defect detection, owing to the limited representation capacity of conventional methods in complex backgrounds [7]. For magnetic tile defect localization and feature extraction, early studies directly adopted general object detectors, including Faster region-based convolutional neural network (R-CNN) [17], Mask R-CNN [18], Detection Transformer (DETR) [19], Deformable DETR [20] and RT-DETR [21]; however, deep downsampling can readily discard small defect features, particularly for small and low-contrast defects. To address the difficulty of magnetic tile feature extraction, Xie et al. [22] proposed the Fusion Feature convolutional neural network (FFCNN), and Liang et al. [23] developed the feature-enhancement loop-shaped fusion convolutional neural network, which filters complex magnetic tile backgrounds through multiscale feature extraction and substantially strengthens defect features. Zhu et al. [24] designed a CNN with rotation invariance to handle fine defects that are randomly distributed on magnetic tiles. In addition, Du et al. [25] proposed the adaptive focusing feature network (AFF-Net) for strip steel surface defect detection, which employs adaptive focusing to enhance the localization of faint defects on complex industrial surfaces. Liu et al. [26] proposed a global attention module and cascade fusion network to improve the handling of unstructured defects, while Yan et al. [27] developed the Global Context Guided Refinement and Aggregation Network, which captures global contextual information through cross-dimensional attention across channel and spatial dimensions.

For pixel-level segmentation of magnetic tile defects, U-Net [28], DeepLabV3+ [29] and CCNet [30] have also been applied. To address class imbalance in magnetic tile images, Huang et al. [31] designed a multi-scale fusion-staged U-shaped convolutional neural network, which alleviates missed segmentation caused by variable defect morphologies. Liu et al. [32] proposed an adaptive image segmentation network to capture rich local details through dual attention, and Cao et al. [33] developed an attention-enhanced flexible U-Net to further improve segmentation accuracy.

Although segmentation networks continue to improve, the low contrast of magnetic tiles remains a key limitation on detection accuracy. Researchers have therefore introduced feature enhancement mechanisms. Cui et al. [34] proposed a joint framework combining semantic discrimination and contrast transformation, which dynamically refines weak defect representations, and Du et al. [35] employed generative adversarial networks to augment limited feature data. Meanwhile, Zhang et al. [36] proposed Locally Adaptive Contrast Enhancement (LACE), which dynamically adjusts contrast based on the local statistical variance of the image, offering an effective means of enhancing faint defect features in magnetic tiles. However, random stains and noise artefacts covering magnetic tile surfaces mean that conventional global enhancement risks indiscriminate noise amplification. Accordingly, co-salient object detection has emerged as a complementary solution. Fan et al. [37] proposed the group collaborative learning framework, which suppresses complex background interference by mining consensus representations in feature space. Building on this work, Zhu et al. [38] proposed Co-Representation Purification (CoRP), which searches for a noise-free co-representation. These commonality-mining methods provide an effective means of recovering magnetic tile defects under low contrast; nevertheless, they have predominantly been applied to natural image datasets and their integration with task-specific defect localization and local contrast enhancement remains largely unexplored, which motivates the three-stage framework proposed in this work.

Specifically, related multi-stage methods combine region selection with segmentation refinement. Wang et al. [39] proposed LPC-Net, which guides segmentation through defect localization and refines predictions using neighborhood pixel confidence. Chen et al. [40] combined image-level suspected-defect filtering with pixel-level segmentation. In biomedical imaging, Benčević et al. [41] introduced Segment-then-Segment, which uses coarse segmentation to extract context-preserving crops for subsequent refinement. Building on these region-focused strategies, the proposed framework further integrates local contrast enhancement and cross-image co-representation purification with frequency-aware localization. This integration not only reduces background dominance through ROI extraction but also improves local defect visibility and suppresses residual background interference within the selected regions.

Despite these advances, jointly addressing spatial sparsity, low contrast, and complex background textures remains challenging in magnetic tile inspection. Region selection reduces irrelevant background but does not directly resolve weak boundaries or distracting textures within an ROI. The research gap therefore lies in coordinating region selection with local enhancement and foreground discrimination. The proposed framework addresses this gap by linking these complementary functions in a three-stage pipeline.

## 3. Proposed Method

**Figure 2:**
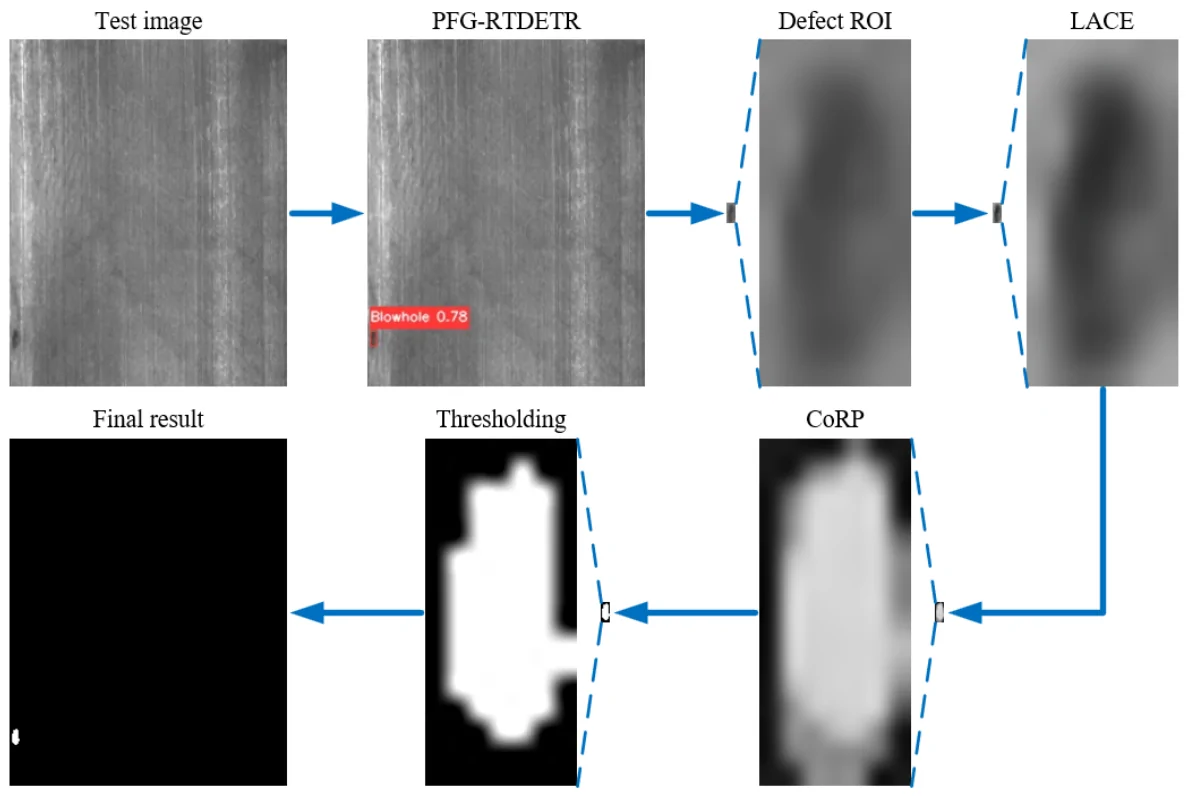
Overall flowchart.

We propose a localization-enhancement-segmentation detection framework, comprising defect localization based on PFG-RTDETR, defect contrast enhancement based on LACE, and defect segmentation based on CoRP. The overall flowchart is shown in Figure 2.

### 3.1. Defect Localization Based on PFG-RTDETR

#### 3.1.1. Overall Structure of the RT-DETR Algorithm

**Figure 3:**
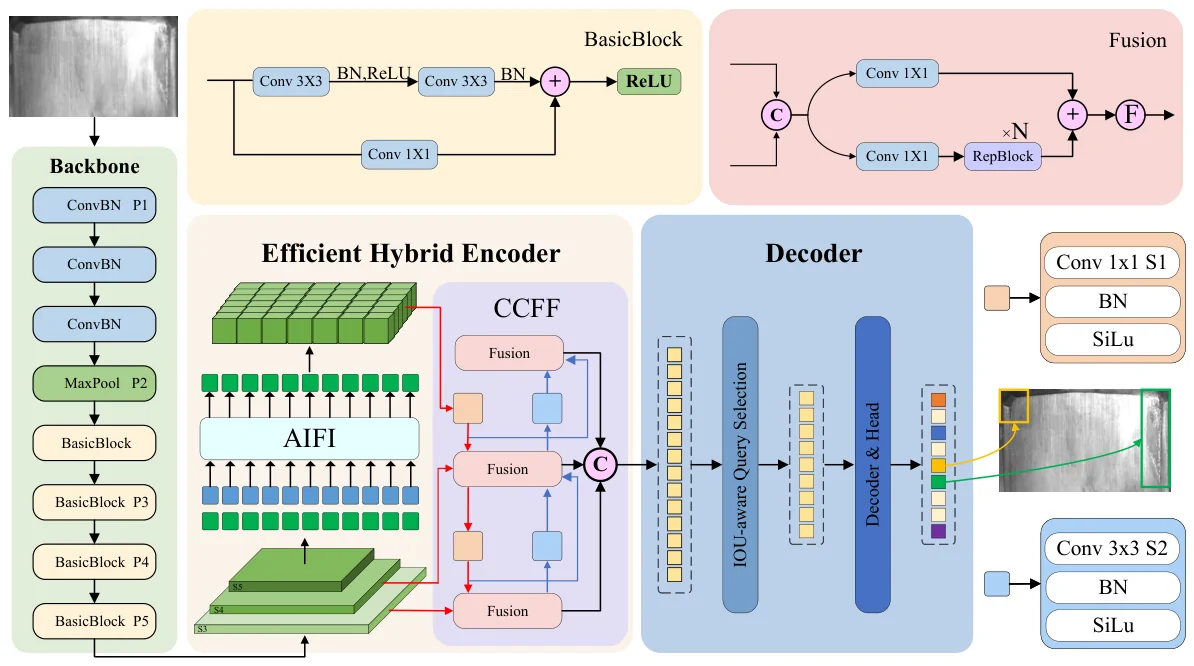
RT-DETR model framework.

RT-DETR [21] is an advanced end-to-end object detector that provides a favourable speed-accuracy trade-off relative to the You Only Look Once (YOLO) series. By removing conventional non-maximum suppression (NMS) and employing optimised post-processing, RT-DETR reduces redundant detections and avoids missed and false detections caused by NMS threshold sensitivity in magnetic tile images with low contrast. This makes it well-suited for magnetic tile inspection scenarios with high requirements for defect localization accuracy. We use ResNet18 as the baseline. As shown in Figure 3, it consists of a backbone, an efficient hybrid encoder, and a decoder.

The backbone uses convolutional layers to extract hierarchical features. The input image passes through convolution, max pooling, and several basic blocks to produce feature maps P3, P4, and P5 at strides of 8, 16, and 32, respectively. The efficient hybrid encoder consists of Attention-based Intra-scale Feature Interaction (AIFI) and CNN-based Cross-scale Feature Fusion (CCFF). AIFI is applied only to the deepest feature map P5 to capture global feature interactions, while CCFF fuses P5, P4, and P3 through top-down and bottom-up paths. In the decoder, the three CCFF output feature maps are flattened, concatenated, and fed to a Transformer-based denoising decoder, which improves positive query quality and accelerates convergence. Finally, the model employs a dynamic intersection over union (IoU) allocation strategy to select a fixed number of object queries and iteratively refines their locations and class predictions.

#### 3.1.2. Structure of the Improved PFG-RTDETR Algorithm

**Figure 4:**
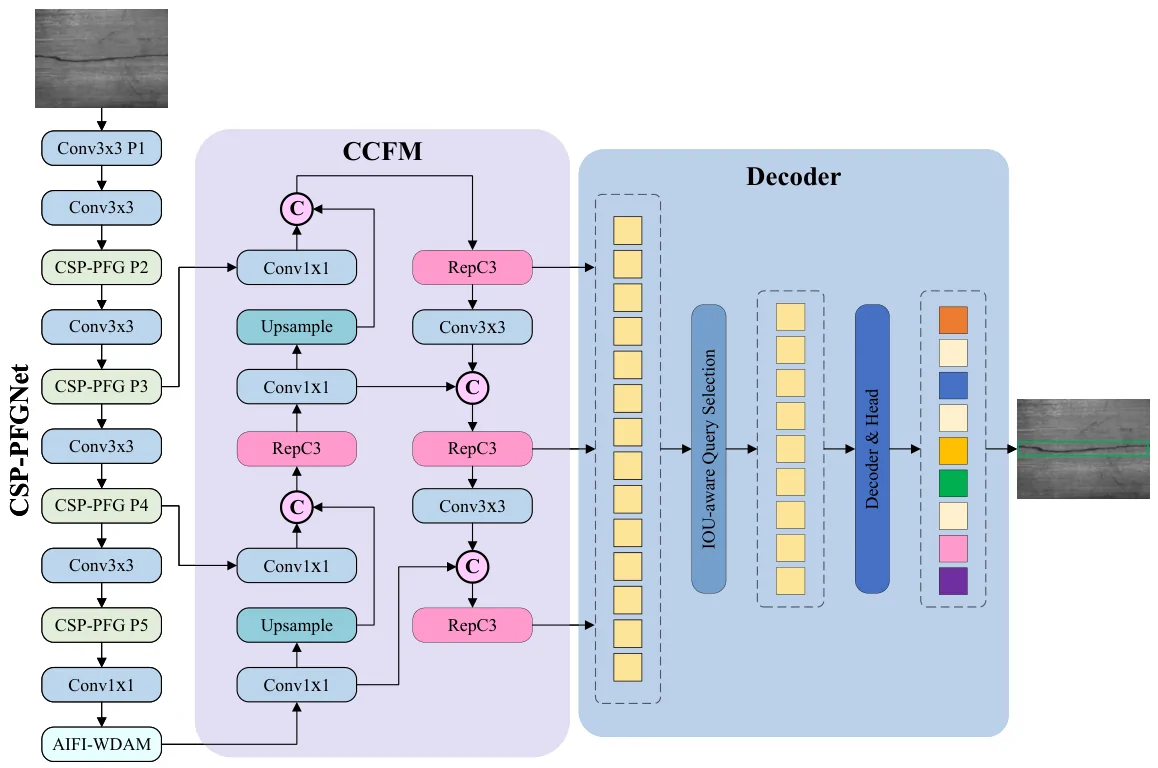
PFG-RTDETR model.

The improved model, termed PFG-RTDETR, is shown in Figure 4. Like RT-DETR, it comprises a backbone, a hybrid encoder, and a decoder. Because conventional feature extraction methods are inadequate for the complex task of detecting magnetic tile surface defects, PFG-RTDETR incorporates the proposed CSP-PFGNet backbone and AIFI-WDAM module. CSP-PFGNet enhances defect feature extraction, whereas AIFI-WDAM suppresses interference from complex background noise on magnetic tile surfaces. Together, they markedly improve defect recognition accuracy and the extraction of edge details under low-contrast conditions.

(1) CSP-PFGNet backbone

**Figure 5:**
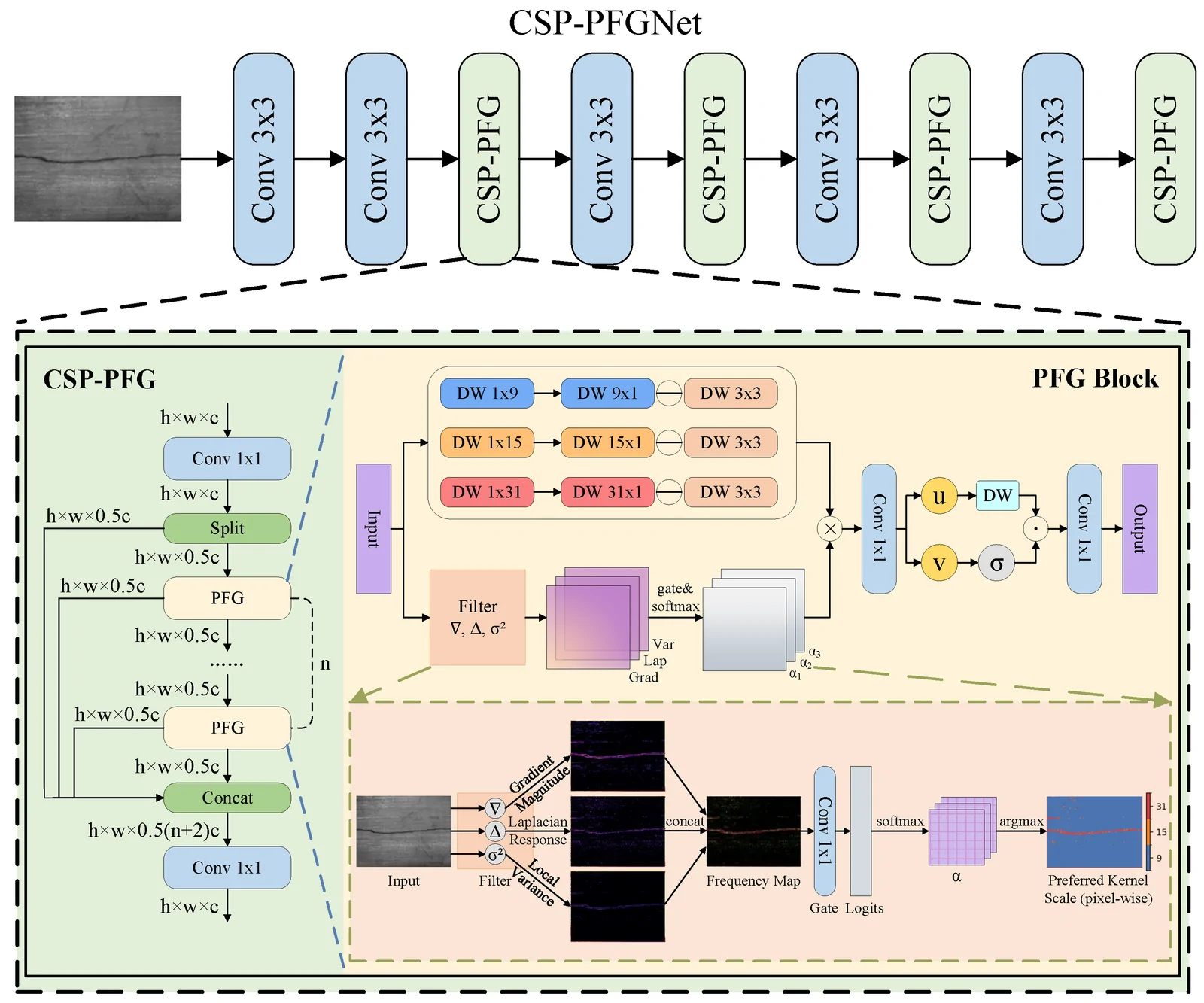
CSP-PFGNet architecture diagram.

ResNet18 has a fixed convolutional architecture and is insufficiently adaptable to multi-scale and fine-grained information, which limits its performance on magnetic tile defect detection. To address this, inspired by Cross Stage Partial (CSP) connections [42] and Peripheral Frequency Gating (PFG) [43], we design CSP-PFGNet to replace ResNet18 in RT-DETR [44]. CSP-PFGNet contains two core modules: (a) a CSP-PFG cross-stage feature-propagation module and (b) a PFG frequency-guided gating feature extraction module. The former improves feature propagation through cross-stage splitting and fusion, while the latter enables precise defect localization through frequency-guided gating. As shown in the upper part of Figure 5, CSP-PFGNet alternates 3 × 3 convolutions and CSP-PFG blocks. The convolutions perform feature extraction and downsampling, while CSP-PFG blocks enhance deep features. This design preserves small defect information during progressive downsampling and reduces missed detections. The lower part of Figure 5 expands the two core modules, CSP-PFG and PFG, which perform feature propagation and feature extraction, respectively.

(a) CSP-PFG Feature Propagation Module. As shown in the expanded view in Figure 5, CSP-PFG improves feature flow through cross-stage propagation. It splits the input features into two branches: the main branch is forwarded directly, whereas the other branch enters a deep path containing several PFG blocks. Rather than processing every pixel indiscriminately as conventional convolutions do, the PFG blocks employ pixel-wise frequency guidance to extract multi-dimensional local frequency cues, combine these with multi-scale separable convolutions to expand the receptive field, and introduce centre suppression to reduce background noise interference. These operations collectively improve defect localization accuracy. CSP-PFG then fuses the frequency-refined deep features with the unmodified features forwarded by the main branch. The resulting representation retains global structure and fine detail, reduces the parameter count, and suppresses complex-background interference, thereby improving detection performance and edge-extraction accuracy in low-contrast images.

(b) PFG Feature Extraction Module. Within CSP-PFGNet, the PFG block actively attends to defects and adaptively suppresses the background early in feature extraction. As shown in the expanded PFG block in Figure 5, it comprises three stages: pixel-wise frequency-guided gating, multi-scale peripheral response with centre suppression, and channel feature mixing. Pixel-wise frequency guidance derives multi-dimensional frequency cues from gradient magnitude, Laplacian response, and local variance to produce spatial attention weights for defects with different morphologies. Multi-scale peripheral response with centre suppression enhances defects and suppresses background noise through the dynamic difference between peripheral large-kernel convolutions and a central penalty term. Channel feature mixing employs gated linear units to fuse multi-scale features and exchange information across channels. Overall, the input features are first split into upper and lower branches. The lower branch extracts image frequency information through frequency guidance, whereas the upper branch combines multi-scale large-receptive-field convolutions (1 × 9, 1 × 15, and 1 × 31) with a central 3 × 3 convolution to extract structural features and suppress noise. Finally, the two branch features are fused with weights and passed through channel mixing to produce the enhanced feature map.

Specifically, the theoretical implementation of the three core steps is as follows:

(i) Pixel-wise frequency-guided gating. This step constitutes a frequency-domain spatial-attention generator that assigns each pixel weights for receptive fields of different sizes based on multiple local frequency features, thereby enabling precise localization of small defects with different morphologies.

Different defects in magnetic tile images possess distinct physical characteristics. Cracks exhibit continuous directionality, whereas blowholes have closed contours. Breaks and frays correspond to abrupt edge changes or texture degradation, while background noise appears as unstructured, random high-frequency components. Based on this physical rationale, given the input feature *X*, the module extracts local frequency information from three perspectives: gradient magnitude, Laplacian response, and local variance. For a multi-channel feature *X*, the three frequency cues are computed independently for each channel using fixed depthwise filters and then averaged along the channel dimension to obtain single-channel frequency maps.

First, using the horizontal and vertical Sobel operators $G_{x}$ and $G_{y}$, the gradient magnitude $f_{1}$ of the feature map is calculated; this first-order spatial derivative captures the edges of cracks with continuous directionality on the surface of the magnetic tiles:

(1) $$f_{1}=\sqrt{{\left(G_{x}*X\right)}^{2}+{\left(G_{y}*X\right)}^{2}}$$

Second, the absolute value of the second-order derivative response is calculated using the Laplacian operator *L*, yielding $f_{2}$. The second-order derivative is sensitive to abrupt changes in curvature and can accurately identify minute blowholes with locally closed contours:

(2) $$f_{2}=\left|L*X\right|$$

Third, the texture complexity features of the image are obtained via local variance $f_{3}$, which are used to identify local break and fray defects. Here, $E_{3\times 3}\left[\cdot \right]$ denotes the local mean pooling operation of 3×3:

(3) $$f_{3}=E_{3\times 3}\left[X^{2}\right]-{\left(E_{3\times 3}\left[X\right]\right)}^{2}$$

The three extracted frequency features are concatenated to form a frequency feature description map $F=[f_{1},f_{2},f_{3}]$, which is then mapped by a 1×1 convolution to the selection logits $Z_{g}$ for the receptive-field scales:

(4) $$Z_{g}=W_{g}*F+b_{g}$$

where $W_{g}$ and $b_{g}$ denote the learnable weights and biases, respectively. Finally, a *Softmax* activation function is applied along the scale dimension *K* to generate per-pixel gating weights $\alpha_{k}$ for different scale receptive fields:

(5) $$\alpha_{k}\left(h,w\right)=\frac{\exp(Z_{g,k}\left(h,w\right))}{\sum_{j=1}^{K}\exp(Z_{g,j}\left(h,w\right))}$$

where *K* is the specified number of receptive-field branches. This mechanism adaptively assigns scale-specific receptive field weights to each pixel, enabling precise spatial focusing of feature extraction.

(ii) Multi-Scale Peripheral Response with Center Suppression. This mechanism employs ultra-large-scale one-dimensional convolutions of sizes 1 × 9, 1 × 15, and 1 × 31 to extract extensive continuous spatial features, and applies centre suppression and differential operations to highlight structural defects whilst suppressing random noise.

Because both defects and noise manifest as high-frequency components in magnetic tile images with low contrast, the PFG block introduces multi-scale peripheral receptive fields K={9,15,31} and exploits geometric correlations to distinguish them. It decomposes large two-dimensional convolutions into separable one-dimensional convolutions to reduce computational overhead and computes the corresponding scale-specific peripheral responses $P_{k}$:

(6) $$P_{k}=v_{k}*\left(h_{k}*X\right)$$

where $k\in K,\;h_{k}\in R^{1\times k}$ and $v_{k}\in R^{k\times 1}$ represent the horizontal and vertical convolution kernels, respectively, used to extract peripheral spatial structural features. A centre suppression mechanism is then introduced to obtain the final output of the scale branch:

(7) $$Y_{k}=P_{k}-\tanh\left(\beta_{k}\right)⊙\left(C*X\right)$$

Here, $Y_{k}$ represents the purified output features; C∗X denotes the response extracted via the 3×3 convolution; ⊙ denotes element wise multiplication; and $\beta_{k}$ denotes channel-level learnable parameters. The term $-\tanh\left(\beta_{k}\right)⊙\left(C*X\right)$ constitutes the suppression term for the central high-frequency response, in which the tanh function constrains the suppression coefficients to the range [−1, 1], working in conjunction with $\beta_{k}$ to form a bidirectional regulator that prevents excessive amplification of high-frequency noise. When the input consists of random noise, the lack of spatial structural correlations causes $P_{k}$ to tend towards zero, at which point the centre suppression term dominates, thereby achieving denoising. When the input consists of structural defects such as cracks or blowholes, the peripheral receptive fields aggregate contextual information, causing $P_{k}$ to be substantially enhanced and to dominate the output, ultimately preserving and enhancing the defect features in $Y_{k}$.

Subsequently, after extracting multi-scale features, the outputs $Y_{k}$ from branches of different scales are dynamically fused using per-pixel gated weights $\alpha_{k}$:

(8) $$PFG\left(X\right)=\sum_{k\in K}\;\alpha_{k}⊙Y_{k}$$

As the multi-scale feature extraction involves depthwise separable convolutions operating in independent spatial dimensions, a channel mixer is introduced to facilitate cross-channel feature interaction.

(iii) Channel Feature Mixing. Channel feature mixing employs gated linear units to facilitate information exchange between channels, performing nonlinear reorganisation of the fused spatial features along the channel dimension. This compensates for the shortcomings of independent spatial convolutions and enhances the nonlinear expressive power of the features.

The fused features are first projected to a higher channel dimension via a 1×1 convolution, then split equally along the channel dimension into ‘*U*’ and ‘*R*’. The *R* branch undergoes a 3×3 depthwise convolution ($DW_{3\times 3}$) to incorporate local spatial inductive bias, whilst the *U* branch undergoes a *Sigmoid* activation (σ) to generate dynamic gated weights. The two branches are multiplied element-wise to achieve nonlinear reorganisation; finally, dimension reduction via pointwise convolution (PW) yields the output $Z_{c}$:

(9) $$Z_{c}=PW\left(\sigma \left(U\right)⊙DW_{3\times 3}\left(R\right)\right)$$

Through the above design, CSP-PFGNet improves feature propagation and extraction while preserving small defect information during downsampling. Specifically, the CSP-PFG module optimises feature flow through cross-stage splitting and fusion, while the PFG module employs pixel-wise frequency-guided gating and centre suppression to adaptively capture defect features and suppress background noise, thereby addressing the weak representation of small and multi-scale defects that are easily obscured or lost in complex backgrounds.

(2) AIFI-WDAM Module

**Figure 6:**
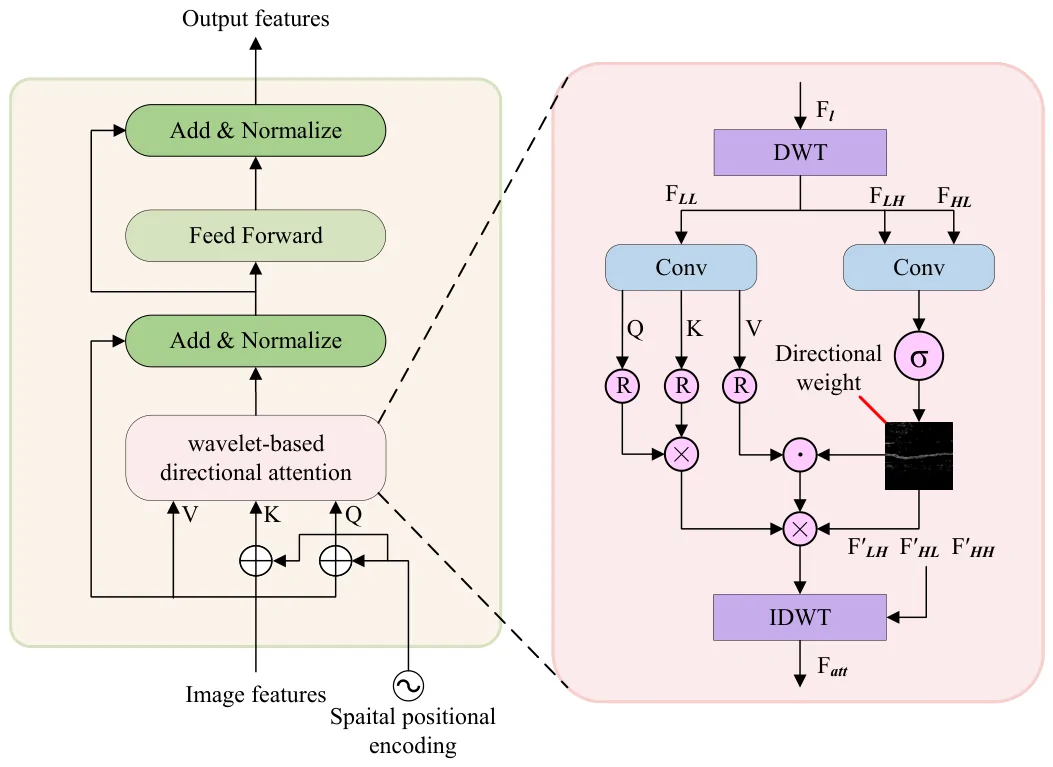
AIFI-WDAM architecture diagram.

Against a large-area magnetic tile background, the AIFI in the RT-DETR hybrid encoder applies global self-attention that treats all pixels equally; this tends to cause weak defect features to be diluted by background information, thereby reducing detection accuracy. To address this, we introduce a wavelet-based directional attention module (WDAM) [45] into AIFI, proposing a frequency-domain-guided intra-scale feature interaction layer termed AIFI-WDAM, whose network architecture is shown in Figure 6. This module employs wavelet transform to decouple and extract image signals by frequency band and spatial direction, thereby breaking the uniform weighting mechanism of self-attention through directional priors. Specifically, deep image features serve as value vectors *V*. Concurrently, these features are combined with positional encoding via element-wise addition (⨁) to yield the query vector *Q* and key vector *K*, before jointly entering the wavelet-based directional attention sublayer for frequency-domain interaction. Finally, after residual connection and normalisation, the output is fed into a feedforward network to yield an encoded representation that incorporates both global and fine-grained information.

In AIFI-WDAM, the WDAM module is responsible for global feature interaction. As illustrated by the expanded WDAM module on the right side of Figure 6, when processing deep-layer input features $F_{l}$, the module employs discrete wavelet decoupling to construct a dual-branch processing architecture, dividing the features into four subbands for differentiated processing.

First, the Haar discrete wavelet transform (DWT) linearly decomposes $F_{l}$ into four subbands in the spatial domain:

(10) $$\left[F_{LL},F_{LH},F_{HL},F_{HH}\right]=DWT\left(F_{l}\right)$$

Here, $F_{LL}$ represents the low-frequency subband, obtained by horizontal and vertical low-pass filtering to capture the large-scale smooth background. $F_{LH}$ and $F_{HL}$ are the horizontal and vertical high-frequency subbands, respectively, which extract edges and gradient changes in the horizontal and vertical directions. $F_{HH}$ is the diagonal high-frequency subband, which captures diagonal edges and abrupt changes via high-pass filtering.

In the low-frequency global interaction branch, to reduce computational complexity and avoid interference from high-frequency noise, global attention calculations are performed only on the low-frequency subband $F_{LL}$. After $F_{LL}$ is projected via a convolutional layer, a *Reshape* operation generates the query matrix *Q*, the key matrix *K*, and the value matrix *V*.

In the high-frequency directional weight branch, the module focuses on spatial geometric correlations and directional continuity. For instance, tiny cracks typically extend along horizontal or vertical directions, generating continuous responses in $F_{LH}$ and $F_{HL}$. Random noise, however, lacks directional coherence and predominantly manifests as discrete extrema in $F_{HH}$. To eliminate noise interference, $F_{HH}$ is excluded when constructing directional weights; instead, $F_{LH}$ and $F_{HL}$, which capture structural defect representations, are concatenated along the channel dimension. Directional structural features are subsequently extracted via a 3×3 convolution, and a *Sigmoid* activation function (σ) is applied to yield the directional weight mask *M*:

(11) $$M=\sigma (Conv\left(\left[F_{LH},F_{HL}\right]\right))$$

As depicted in the directional weight feature map in Figure 6, *M* highlights prominent defect regions by assigning response weights approaching 1, while suppressing noise and homogeneous background areas with near-zero weights.

Subsequently, within the attention calculation, the directional weight mask *M* is element-wise multiplied (⊙) with the value matrix *V*. The resulting features are then combined with the similarity matrix computed from *Q* and *K* via dot product (⊗), alongside a relative positional bias *B*, to yield the final weighted output:

(12) $$Att\left(Q,K,V,M\right)=Softmax\left(\frac{QK^{T}}{\sqrt{d}}+B\right)\left(M⊙V\right)$$

where *d* represents the feature dimension of each attention head, and *B* denotes a learnable relative positional bias introduced prior to the Softmax operation. The bias *B* enables the attention distribution to incorporate relative spatial relationships between feature positions alongside their semantic similarity. In magnetic tile defect inspection, this positional information aids in preserving the spatial continuity and structural integrity of defect features during deep feature interaction. Furthermore, through modulation via the mask *M*, the extensive low-frequency background that originally dominated the *V* matrix is effectively suppressed, while defect signals with continuous directionality are enhanced. Consequently, the network focuses attention on structural feature regions, yielding purified low-frequency features, denoted as $F_{LL}^{\prime }$, that effectively mitigate oversmoothing.

Finally, $F_{LL}^{\prime }$ is fused with the refined high-frequency subbands ($F_{LH}^{\prime }$, $F_{HL}^{\prime }$, and $F_{HH}^{\prime }$); the spatial features are subsequently reconstructed via the inverse discrete wavelet transform (IDWT) to yield high-dimensional output representations at the original spatial resolution, denoted as $F_{att}$:

(13) $$F_{att}=IDWT\left(F_{LL}^{\prime },F_{LH}^{\prime },F_{HL}^{\prime },F_{HH}^{\prime }\right)$$

Through this design, AIFI-WDAM preserves the global modeling capability of the Transformer while leveraging wavelet-based directional attention to suppress the oversmoothing effect induced by the background on subtle defects, thereby markedly enhancing defect recognition robustness under complex, noisy environments.

### 3.2. Defect Contrast Enhancement Based on LACE

The inherently low contrast of magnetic tile images hinders the discrimination of defect features. We therefore crop defect regions of interest (ROIs) using the bounding coordinates predicted by PFG-RTDETR and apply Locally Adaptive Contrast Enhancement (LACE) [36] within each ROI. LACE adaptively modulates pixel intensity according to local image statistics, suppressing background noise while selectively boosting weak defect signals. Crucially, this localized operation prevents subtle features from being attenuated or erased during global denoising.

Specifically, LACE is formulated based on local mean and variance statistics. First, to satisfy real-time industrial requirements, an integral image S(x,y) is introduced to accelerate feature computation. For a given ROI image *I*, the integral image enables O(1) constant-time calculation of the pixel sum within any window Ω centred at (x,y) (i.e., the execution time is independent of the window size *w*), thereby substantially boosting computational efficiency. Here, I(i,j) denotes the grayscale value of the pixel at coordinate (i,j) within the ROI image. The integral image is defined as:

(14) $$S\left(x,y\right)=\sum_{i\leq x,j\leq y}\;I\left(i,j\right)$$

To estimate local statistics for a window of size w×w centred at (x,y), the local mean μ(x,y) and local standard deviation σ(x,y) are calculated according to Equations (15) and (16), respectively:

(15) $$\mu \left(x,y\right)=\frac{1}{w^{2}}\sum_{(i,j)\in \Omega }\;I\left(i,j\right)$$

(16) $$\sigma \left(x,y\right)=\sqrt{\frac{1}{w^{2}}\sum_{(i,j)\in \Omega }\;\;I^{2}\left(i,j\right)-\mu^{2}\left(x,y\right)}$$

Subsequently, based on local statistics, the local mean is approximated as the low-frequency component; the high-frequency component is extracted by subtracting the mean from the original pixel intensities; and an enhancement control factor λ [36] is introduced to amplify high-frequency details and boost image contrast. The enhanced output pixel intensity J(x,y) is defined in Equation (17):

(17) J(x,y)=μ(x,y)+λ·[I(x,y)−μ(x,y)]

Following LACE processing, defect contours within each ROI exhibit enhanced clarity with sharpened boundaries, while the background is effectively smoothed. Consequently, LACE highlights subtle defect features in low-contrast magnetic tile images, providing more discriminative representations for subsequent segmentation.

### 3.3. Defect Segmentation Based on CoRP

Defect segmentation is subsequently executed on the locally enhanced, high-contrast magnetic tile ROIs. However, because the localization and enhancement stages cannot fully suppress random noise, single-image segmentation remains vulnerable to artifacts, boundary degradation, under-segmentation, and mis-segmentation. To overcome these constraints, we observe that while an individual sample under low-contrast conditions often exhibits ill-defined boundaries, defects belonging to the same category display intrinsic co-saliency within the feature space. Consequently, co-salient object detection can be leveraged to mine these shared attributes, thereby boosting overall segmentation precision.

However, existing feature aggregation methods typically extract co-representations globally across the entire image, which remains susceptible to noise corruption. To mitigate this limitation, we incorporate the Co-Representation Purification (CoRP) network [38]. Guided by a “less is more” selection rationale, CoRP selectively retains only the most reliable co-representation embeddings for feature purification. For instance, in porosity defects with consistent geometric structures, feature representations from high-quality exemplars within the group effectively complement localized boundary details in images degraded by noise or specular reflection. During training and inference, CoRP jointly processes a group of related images to exploit recurring foreground features and suppress image-specific background interference. In the proposed magnetic tile inspection framework, the inputs to CoRP are locally enhanced defect ROIs extracted by the preceding stages. These ROIs are grouped according to the defect categories predicted by the localization stage, enabling CoRP to exploit shared defect characteristics within each group. Mixing unrelated defect categories may weaken this commonality. When only a single input image is available and no additional related images or reference ROIs are provided, cross-image information is unavailable, and the segmentation performance under this condition remains to be evaluated. For images arriving individually in an industrial system, grouping the ROI extracted from an incoming image with previously collected similar defect ROIs offers a potential deployment strategy.The proposed method comprises two key components: Recurrent Proxy Purification (RPP) and Pure Co-Representation Search (PCS), detailed as follows.

First, a feature centre proxy is generated via RPP. Given a batch of *N* magnetic tile images, a corresponding feature collection $F={\left\{F_{n}\right\}}_{n=1}^{N}$ is extracted. To capture shared defect attributes, RPP establishes a closed-loop iterative feedback mechanism to refine the feature centre. Specifically, in the *t*-th iteration, the co-saliency map $M^{t-1}={\left\{M_{n}^{t-1}\right\}}_{n=1}^{N}$ predicted from the preceding iteration serves as an attenuation mask to reweight the original feature set F. This yields a proxy representation $p^{t}\in R^{D}$ that encodes the common defect characteristics across the image set, as defined in Equation (18):

(18) $$p^{t}=\frac{1}{N}\sum_{n=1}^{N}\;GAP\left(M_{n}^{t-1}⊙F_{n}\right)$$

where ⊙ denotes element-wise multiplication and GAP(·) represents global average pooling. As the iteration progresses, the proxy representation $p^{t}$ progressively filters out background noise and converges toward the true centre of the defect representations.

To mitigate the residual random noise present in the direct-averaged proxy $p^{t}$, the PCS module is employed for feature filtering. Specifically, PCS suppresses irrelevant background interference across all pixel embeddings ${\left\{F^{\left(l\right)}\in R^{D}\right\}}_{l=1}^{L}$ by computing the dot-product similarity between each embedding $F^{\left(l\right)}$ and the current proxy $p^{t}$, thereby isolating the most reliable defect representations:

(19) $$Score^{t(l)}=p^{t}\cdot F^{(l)⊤}$$

To obtain a purified feature representation, the similarity scores are sorted in descending order, and the spatial indices ${\mathrm{Index}}^{t}$ corresponding to the top-*K* scores (e.g., K=32) are retained:

(20) $$Index^{t}=argtop_{k}^{\left(l\right)}\left(Score^{t(l)}\right)$$

where argtopK(·) denotes the operation that extracts the spatial indices corresponding to the *K* highest similarity scores.

Subsequently, utilizing these spatial indices, the *K* most purified embedding vectors are retrieved from the feature space to construct the co-representation matrix for the current iteration *t*, denoted as $C^{t}\in R^{K\times D}$:

(21) $$C^{t}=gather\left(F,Index^{t}\right)$$

where gather(·) denotes the operator that collects the corresponding embedding vectors from F according to the spatial indices ${\mathrm{Index}}^{t}$. This step effectively decouples background noise from defect features at the representation level. Once the purified co-representation $C^{t}$ is obtained, it serves as a filter bank to transform each original feature map $F_{n}$ into a tensor of correlation maps $A_{n}^{t}\in R^{K\times H\times W}$, thereby guiding the model to focus on intra-group defect consistency, as defined in Equation (22):

(22) $$A_{n}^{t}=C^{t}\left(\left(p^{t}\cdot F_{n}\right)⊙F_{n}\right)$$

This correlation mapping enables the network to leverage structural information from high-quality exemplars within the group, thereby reconstructing defect boundaries that are degraded in the target image due to environmental fluctuations or noise interference.

Finally, the decoder processes the set of correlation maps $A^{t}={\left\{A_{n}^{t}\right\}}_{n=1}^{N}$ to generate a more accurate co-saliency map $M^{t}$ for the current iteration; $M^{t}$ is subsequently fed back into the RPP module to compute a more refined proxy representation $p^{t+1}$ for the next cycle. After T=3 closed-loop iterations (empirically determined [38]), artifacts in the predicted maps are progressively suppressed, yielding the optimal purified saliency map.

The refinement achieved by CoRP operates within the ROIs supplied by the preceding localization and enhancement stages. Accordingly, the influence of localization errors on the final segmentation depends on the coverage and content of these ROIs. Moderate bounding-box deviations do not necessarily produce corresponding segmentation errors, provided that the defect remains fully covered and sufficiently resolved. Within such ROIs, LACE enhances local contrast, while CoRP distinguishes defect pixels from the surrounding background, allowing the predicted mask to follow the defect contour rather than the bounding-box boundary. However, an undersized or displaced box may exclude part of a defect, and a defect outside all predicted ROIs receives no subsequent processing. These omissions propagate to the final mask as false-negative pixels, reducing pixel-level recall and IoU. Conversely, an excessively large ROI introduces additional background and reduces the relative defect size after resizing to the fixed segmentation input resolution, potentially weakening fine details. While LACE enhances defect contrast, some background texture responses may also be strengthened. RPP and PCS help distinguish these responses from shared defect features through iterative representation purification, supporting more accurate foreground extraction within the ROIs. Thus, the three stages play complementary roles: localization establishes defect coverage, enhancement improves local visibility, and segmentation refines the pixel-level defect boundaries within the extracted regions.

## 4. Experiments

We evaluated the performance of the proposed method on the benchmark magnetic tile surface defect dataset [3] released by the Institute of Automation, Chinese Academy of Sciences (CASIA), focusing on four primary defect categories: blowholes, cracks, breaks, and frays. The dataset comprises 289 original images with corresponding pixel-level ground-truth masks, including 115 blowhole images, 85 break images, 57 crack images, and 32 fray images. Before data augmentation, the dataset was partitioned into training, validation, and test sets at an approximate ratio of 7:1:2. Subsequently, spatial data augmentation was performed separately within each subset using random rotation, flipping, and cropping, with all augmented images retained in the same subset as their source images. The resulting dataset for localization experiments comprises 2349 images, including 934 blowhole images, 693 break images, 460 crack images, and 262 fray images.

Specifically, the training set contains 81, 40, 59, and 22 original images of blowholes, cracks, breaks, and frays, respectively, together with 577, 283, 422, and 158 additional augmented images, yielding 1642 images in total. The validation set contains 11, 6, 9, and 3 original images and 78, 42, 64, and 22 additional augmented images for the corresponding categories, totaling 235 images. The test set contains 23, 11, 17, and 7 original images and 164, 78, 122, and 50 additional augmented images, totaling 472 images. Although data augmentation increases sample diversity, the limited number of original images and the class imbalance may constrain generalization, particularly for underrepresented defect categories. To further evaluate the applicability of the proposed method to another industrial surface inspection dataset, additional experiments on KolektorSDD2 are presented in the subsequent comparative evaluation.

### 4.1. Defect Localization Experiment Based on PFG-RTDETR

We assessed the effectiveness and robustness of PFG-RTDETR on the magnetic tile dataset, specifically highlighting the individual contributions of CSP-PFGNet and AIFI-WDAM.

#### 4.1.1. Experimental Setup

To ensure reproducibility, all experiments were conducted under a standardized hardware and software environment. The computational setup comprised an Intel Core i5-12600KF CPU, an NVIDIA GeForce RTX 4060 Ti GPU, and Windows 11 OS. The software framework incorporated Python 3.10, CUDA 12.1, and PyTorch 2.2.2. The AdamW optimizer was employed for model training, with an initial learning rate of $1\times 10^{-4}$, regulated by a cosine annealing scheduler.The batch size was set to 4, input images were resized to 640×640 pixels, and training proceeded for 250 epochs. To guarantee a fair comparison, all benchmarked models were trained from scratch without leveraging pre-trained weights.

#### 4.1.2. Evaluation Metrics

To comprehensively evaluate the proposed model, multiple standard performance metrics are adopted. Model complexity is quantified by the parameter count (Params) and giga floating-point operations (GFLOPs), where lower values correspond to reduced computational burden. Detection accuracy is assessed using precision (*P*), recall (*R*), mean average precision at an IoU threshold of 0.50 (${\mathrm{mAP}}_{50}$), and mean average precision integrated over IoU thresholds from 0.50 to 0.95 (${\mathrm{mAP}}_{50–95}$). Higher metric values denote lower rates of false positives and false negatives, reflecting superior localization and recognition capacity.

#### 4.1.3. Results and Discussion

To assess the individual and combined contributions of the proposed modules to magnetic tile defect localization, ablation experiments were performed on the RT-DETR baseline; the quantitative results are detailed in Table 1. Substituting ResNet18 with CSP-PFGNet reduced the parameter count by 26.61% and GFLOPs by 12.46%, while improving ${\mathrm{mAP}}_{50}$ from 89.15% to 90.79%. Integrating AIFI-WDAM independently increased ${\mathrm{mAP}}_{50}$ and ${\mathrm{mAP}}_{50–95}$ to 90.44% and 65.79%, respectively, albeit at the cost of a 15.49% increase in parameter count and a 1.05% increase in GFLOPs. Ultimately, incorporating AIFI-WDAM into CSP-PFGNet elevated ${\mathrm{mAP}}_{50}$ to 90.91% and boosted ${\mathrm{mAP}}_{50–95}$ by 0.94 percentage points over the baseline, while simultaneously reducing parameters and GFLOPs by 11.07% and 11.40%, respectively. Although the combined architecture exhibited a slight 0.11 percentage point decrease in ${\mathrm{mAP}}_{50–95}$ compared to using AIFI-WDAM alone, this minor variation stems from their distinct frequency-selection behaviors. AIFI-WDAM directly leverages directional high-frequency cues, which benefits fine-grained boundary localization under stricter IoU thresholds. Conversely, CSP-PFGNet suppresses low-frequency background interference and random high-frequency noise via the PFG block, which may inadvertently attenuate responses along faint defect boundaries. Nevertheless, the combined model achieved a 0.47 percentage point enhancement in ${\mathrm{mAP}}_{50}$ while compressing parameters from 22.96 M to 17.68 M and GFLOPs from 57.6 G to 50.5 G. These findings validate the complementary synergy between the two modules, enabling PFG-RTDETR to boost detection accuracy while maintaining a lightweight computational footprint.

**Figure 7:**
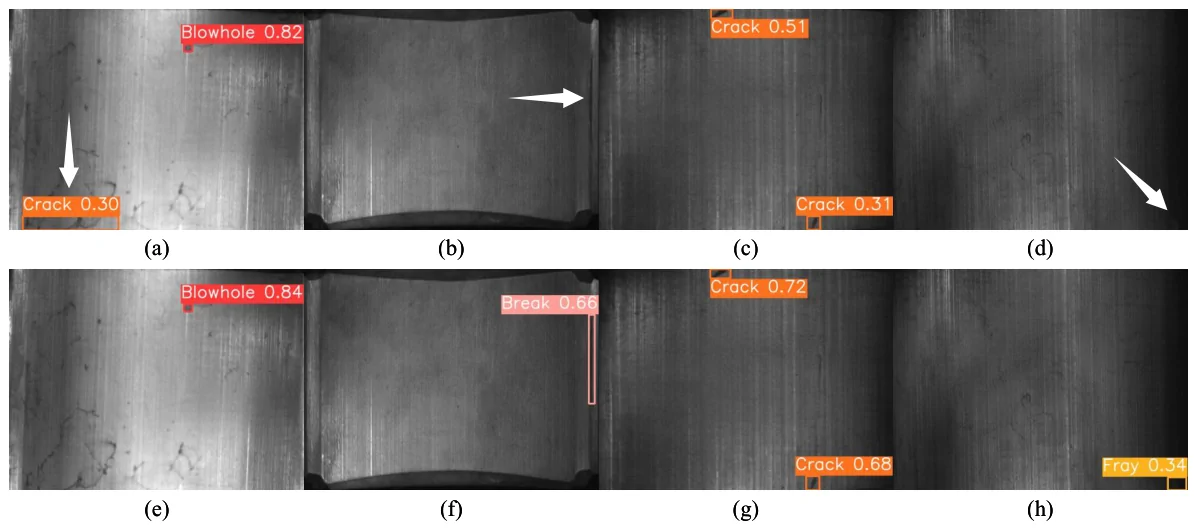
Detection results of (**a**–**d**) RT-DETR and (**e**–**h**) PFG-RTDETR. White arrows indicate a false-positive detection in (**a**) and missed defects in (**b**,**d**).

Figure 7 presents a qualitative visual comparison of localization results between the baseline RT-DETR and PFG-RTDETR. As illustrated in the paired examples (a, e), (b, f), and (d, h), PFG-RTDETR effectively suppresses false alarms and missed detections under challenging low-contrast and noisy background conditions. Furthermore, the paired comparison in (c, g) reveals that PFG-RTDETR boosts the prediction confidence scores for the two crack defects from 0.51 and 0.31 to 0.72 and 0.68, respectively, reflecting substantially improved localization reliability. These visual results validate the efficacy of the proposed frequency-guided gating and wavelet-decoupled directional attention modules, confirming the superior defect localization capability of PFG-RTDETR over the baseline.

Table 2 details the detection performance broken down by defect category. Integrating CSP-PFGNet alone increases the ${\mathrm{mAP}}_{50}$ for blowholes, breaks, and frays by 1.37, 0.68, and 4.55 percentage points, respectively. The most pronounced improvement occurs in the fray category with a gain of 4.55 percentage points, which aligns with its multi-scale frequency-gating mechanism designed to suppress irregular and weakly directional texture degradations. In contrast to the advantage of CSP-PFGNet on irregular textures, AIFI-WDAM exhibits a complementary strength: when incorporated independently, it drives the ${\mathrm{mAP}}_{50}$ for blowholes to a peak of 98.26%, owing to the well-defined and closed contours of blowholes that generate strong directional high-frequency responses. However, because frays lack stable orientation cues, AIFI-WDAM alone achieves a lower ${\mathrm{mAP}}_{50}$ of 82.35% on frays, compared with the 88.19% achieved by CSP-PFGNet.

Although the full PFG-RTDETR model does not achieve the highest AP50 for every defect category, it obtains the highest overall mAP50 of 90.91% among the evaluated configurations. Moreover, as reported in Table 1, it reduces the parameter count from 22.96 M to 17.68 M and the computational cost from 57.6 to 50.5 GFLOPs compared with the AIFI-WDAM-only variant, corresponding to reductions of 23.00% and 12.33%, respectively. These results indicate that, although the individual modules offer advantages for different defect morphologies, their integration provides a favorable balance between overall detection accuracy and computational cost. PFG-RTDETR is therefore selected as the localization component for inspecting diverse magnetic tile defect categories under complex background conditions.

We further assessed the variability of the localization results through three independent training runs for each configuration, using different random seeds while keeping the data split and training settings unchanged. Table 3 reports the mean and sample standard deviation (SD) of each metric, together with the 95% confidence interval (CI) of the mean calculated using Student’s t-distribution with two degrees of freedom.

Table 3 summarizes the localization results across three independent training runs. PFG-RTDETR achieves mean ${\mathrm{mAP}}_{50}$ and ${\mathrm{mAP}}_{50–95}$ values of 90.44% and 65.06%, exceeding the baseline by 1.06 and 1.30 percentage points, respectively. It also exhibits the lowest observed SDs for both metrics, indicating comparatively consistent performance across these runs. Notably, the slight decrease in ${\mathrm{mAP}}_{50–95}$ relative to AIFI-WDAM alone in the original evaluation is not observed in the three-run averages, suggesting that this small single-run difference should be interpreted in the context of training variability. These repeated experiments assess the consistency of the localization results, while the subsequent experiments retain the original pipeline configuration.

**Figure 8:**
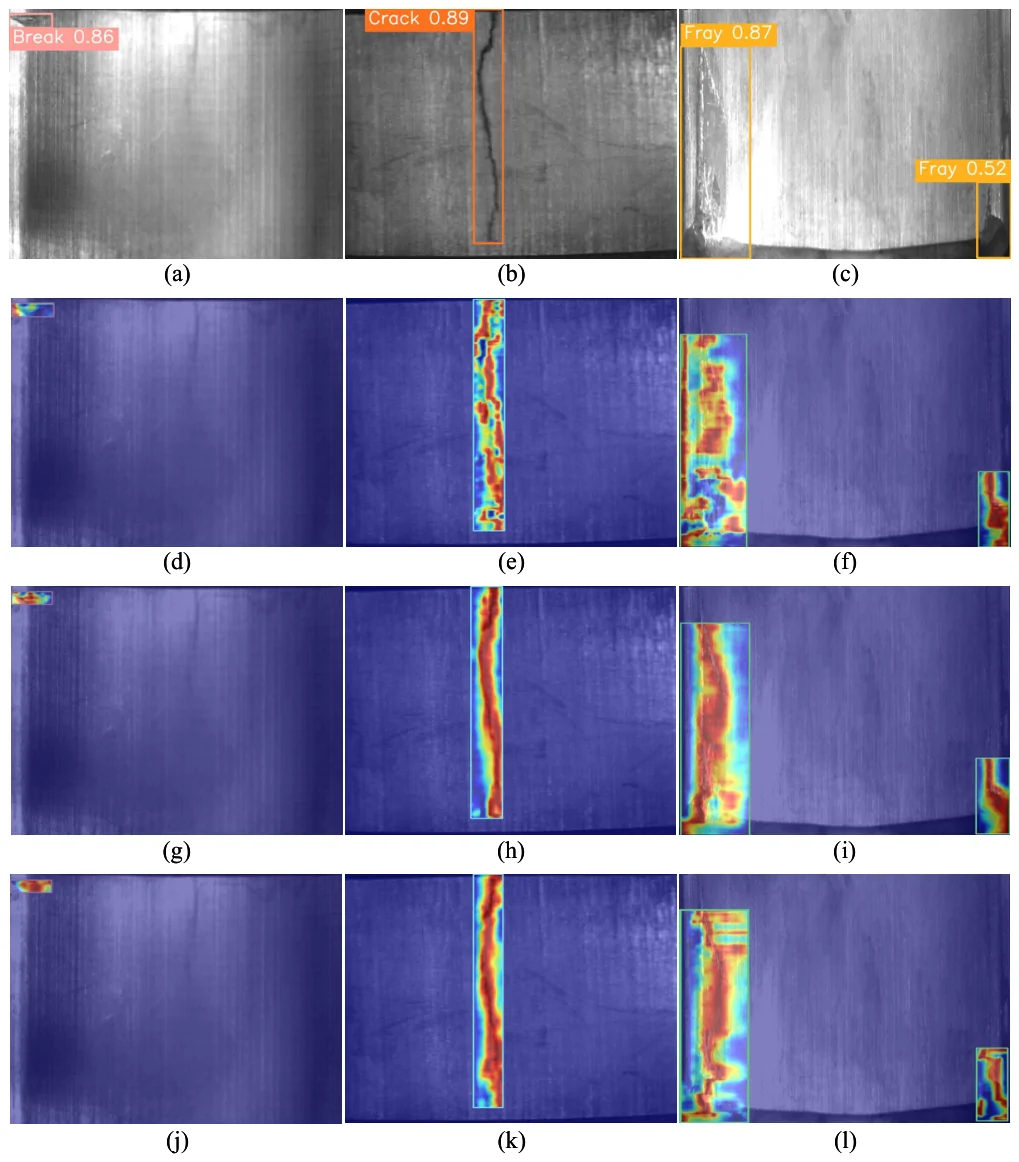
Comparison of feature visualisations before and after module addition. (**a**–**c**) Images with correctly annotated bounding boxes, (**d**–**f**) heatmaps of the original RT-DETR, (**g**–**i**) heatmaps with CSP-PFGNet added, and (**j**–**l**) heatmaps with AIFI-WDAM added. Warmer colors indicate higher Grad-CAM++ activation values, whereas cooler colors indicate lower activation values.

To visually evaluate the feature representation enhancements enabled by CSP-PFGNet and AIFI-WDAM, Gradient-weighted Class Activation Mapping++ (Grad-CAM++) [46] was employed to compare the spatial attention distributions before and after module integration. As illustrated in Figure 8, both modules effectively focus feature attention on actual defect regions. Compared with the baseline RT-DETR (Figure 8d–f), the activation heatmaps for CSP-PFGNet (Figure 8g–i) and AIFI-WDAM (Figure 8j–l) exhibit more pronounced responses over target defects alongside significantly suppressed background interference.

**Figure 9:**
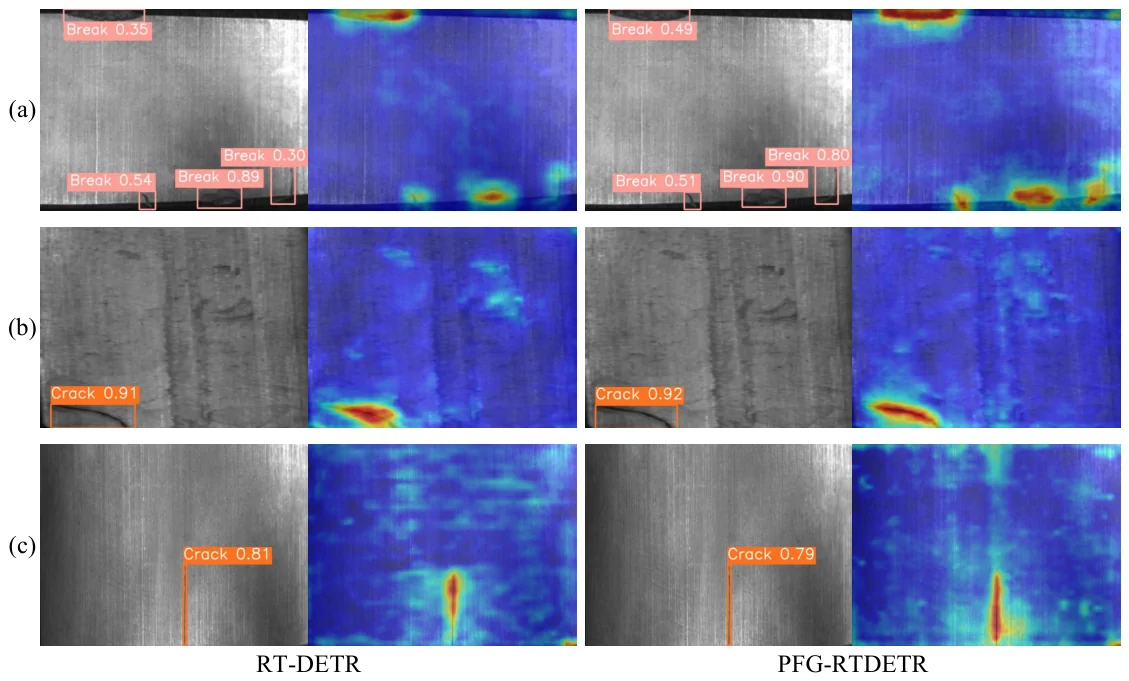
Grad-CAM++ heat-map comparison of RT-DETR and PFG-RTDETR. (**a**) break defects; (**b**) a crack near the lower-left image boundary; (**c**) a thin vertical crack.

To further evaluate the visual interpretability of the proposed model, we compared the feature activation maps of PFG-RTDETR and the baseline RT-DETR using Grad-CAM++, where warmer colors denote higher attention weights toward target regions. As illustrated in Figure 9, PFG-RTDETR generates more concentrated heatmaps that delineate magnetic tile defect regions with higher spatial fidelity across examples (a), (b), and (c), demonstrating a heightened focus on target features. In complex, low-contrast inspection scenes, it captures fine boundary details and subtle texture characteristics more effectively, thereby reinforcing feature representation and boosting both detection accuracy and robustness.

#### 4.1.4. Comparative Experiments

To evaluate the effectiveness of PFG-RTDETR for magnetic tile surface defect localization, YOLOv5s+CRAB, YOLOv4+CRAB [47], and YOLO-RDM [48] were selected as representative baselines. These methods are directly related to magnetic tile defect detection and multi-scale defect localization, which makes them suitable references for evaluating the proposed detector. The convolutional retinal attention block (CRAB) enhances defect representations through multi-resolution feature extraction and global-local attention aggregation, and it is integrated into the YOLOv5s and YOLOv4 detection frameworks. YOLO-RDM combines a residual parallel attention module, depthwise over-parameterized convolution, and a MogaNet backbone to improve multi-scale defect detection in complex backgrounds. In addition, YOLO11m [49], YOLO12m [50], and YOLO26m [51] were included to extend the comparison to recent general-purpose object detectors. YOLO11 improves feature extraction and detection efficiency, YOLO12 adopts an attention-centric architecture, and YOLO26 supports end-to-end detection with an NMS-free inference design. Their medium-sized variants were selected to compare detection accuracy and model complexity with those of PFG-RTDETR. All methods were quantitatively compared using precision, recall, class-wise average precision, overall mean average precision, parameter count, and computational cost.

The quantitative results are reported in Table 4. PFG-RTDETR achieves a precision of 93.98%, exceeding YOLOv5s+CRAB, YOLOv4+CRAB, and YOLO-RDM by 5.37, 4.73, and 5.54 percentage points, respectively. It also outperforms YOLO11m, YOLO12m, and YOLO26m in precision by 4.41, 5.75, and 6.58 percentage points, respectively. These results indicate a lower proportion of false positives among positive predictions. PFG-RTDETR also obtains an mAP_50_ of 90.91%, outperforming YOLOv5s+CRAB, YOLOv4+CRAB, and YOLO-RDM by 2.86, 1.26, and 0.64 percentage points, respectively. Moreover, its mAP_50–95_ reaches 65.68%, corresponding to improvements of 7.35, 9.70, and 4.46 percentage points. Compared with YOLO11m, YOLO12m, and YOLO26m, PFG-RTDETR improves mAP_50_ by 3.22, 4.39, and 3.15 percentage points, and mAP_50–95_ by 5.79, 8.40, and 8.85 percentage points, respectively. The improvements in both metrics indicate better overall detection performance in this evaluation, including performance averaged across multiple intersection-over-union thresholds.

In terms of class-wise average precision, the proposed method achieves the best performance for cracks and frays, reaching 99.50% and 85.92%, respectively. In particular, its average precision for frays exceeds those of YOLOv5s+CRAB, YOLOv4+CRAB, and YOLO-RDM by 7.34, 6.42, and 6.64 percentage points, respectively. he corresponding improvements over YOLO11m, YOLO12m, and YOLO26m are 11.70, 15.62, and 16.74 percentage points, respectively. These results support the effectiveness of the proposed method for crack and fray detection. YOLO11m and YOLO26m achieve the highest AP50 for blowholes and breaks, reaching 98.13% and 88.66%, respectively, whereas PFG-RTDETR obtains lower values for these two categories. Nevertheless, its advantages in crack and fray detection contribute to the highest overall mAP50 among the compared methods, indicating better aggregate detection performance across the four defect categories.

Regarding model complexity, PFG-RTDETR reduces the parameter count and computational cost by 72.97% and 66.45%, respectively, compared with YOLOv4+CRAB. With 17.68 million parameters and a computational cost of 50.5 GFLOPs, PFG-RTDETR also requires fewer parameters and less computation than YOLO11m, YOLO12m, and YOLO26m. Specifically, the parameter count is reduced by 11.86%, 12.21%, and 18.82%, while the computational cost is reduced by 25.95%, 25.52%, and 32.40%, respectively. Together with the higher mAP_50_ and mAP_50–95_, these results demonstrate that PFG-RTDETR achieves a more favorable trade-off between detection accuracy and model complexity than the three medium-sized YOLO models. YOLOv5s+CRAB adopts the lightweight YOLOv5s architecture, whereas YOLO-RDM is developed from the smaller YOLOv8n model. Therefore, both models have fewer parameters and lower computational costs than PFG-RTDETR. However, PFG-RTDETR surpasses these two methods in mAP_50–95_ by 7.35 and 4.46 percentage points, respectively. This advantage under stricter intersection-over-union thresholds indicates more accurate localization and provides more reliable defect ROIs for subsequent local enhancement and fine-grained segmentation.

**Figure 10:**
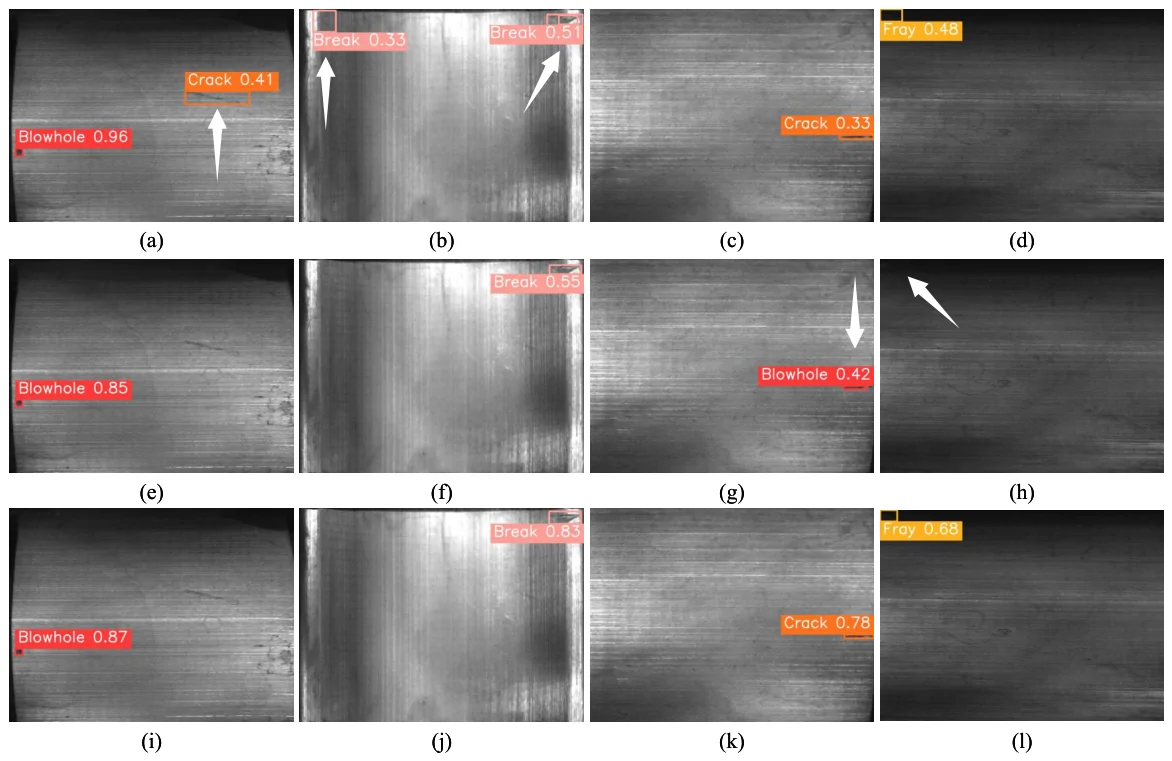
Detection results of (**a**–**d**) YOLOv4+CRAB, (**e**–**h**) YOLOv5s+CRAB, and (**i**–**l**) the proposed PFG-RTDETR. White arrows indicate a false crack detection in (**a**), a false break detection in (**b**), a crack misclassified as a blowhole in (**g**), and a missed fray defect in (**h**).

Figure 10 presents the detection results of the two CRAB-based methods and PFG-RTDETR. For the blowhole and break examples, YOLOv4+CRAB produces an additional false crack detection in Figure 10a and a false break detection in Figure 10b, whereas the proposed method avoids these false responses in Figure 10i,j. For the low-contrast crack, YOLOv4+CRAB yields a detection confidence of only 0.33 in Figure 10c, while YOLOv5s+CRAB misclassifies it as a blowhole in Figure 10g. In contrast, the proposed method correctly identifies the crack with a confidence of 0.78 in Figure 10k. For the fray example, YOLOv5s+CRAB misses the defect in Figure 10h, whereas the proposed method detects it in Figure 10l, with a confidence of 0.68 compared with 0.48 for YOLOv4+CRAB. These qualitative observations are consistent with the quantitative results and further demonstrate the advantages of the proposed method in suppressing background-induced false positives, reducing category misclassification, and detecting subtle defects.

**Figure 11:**
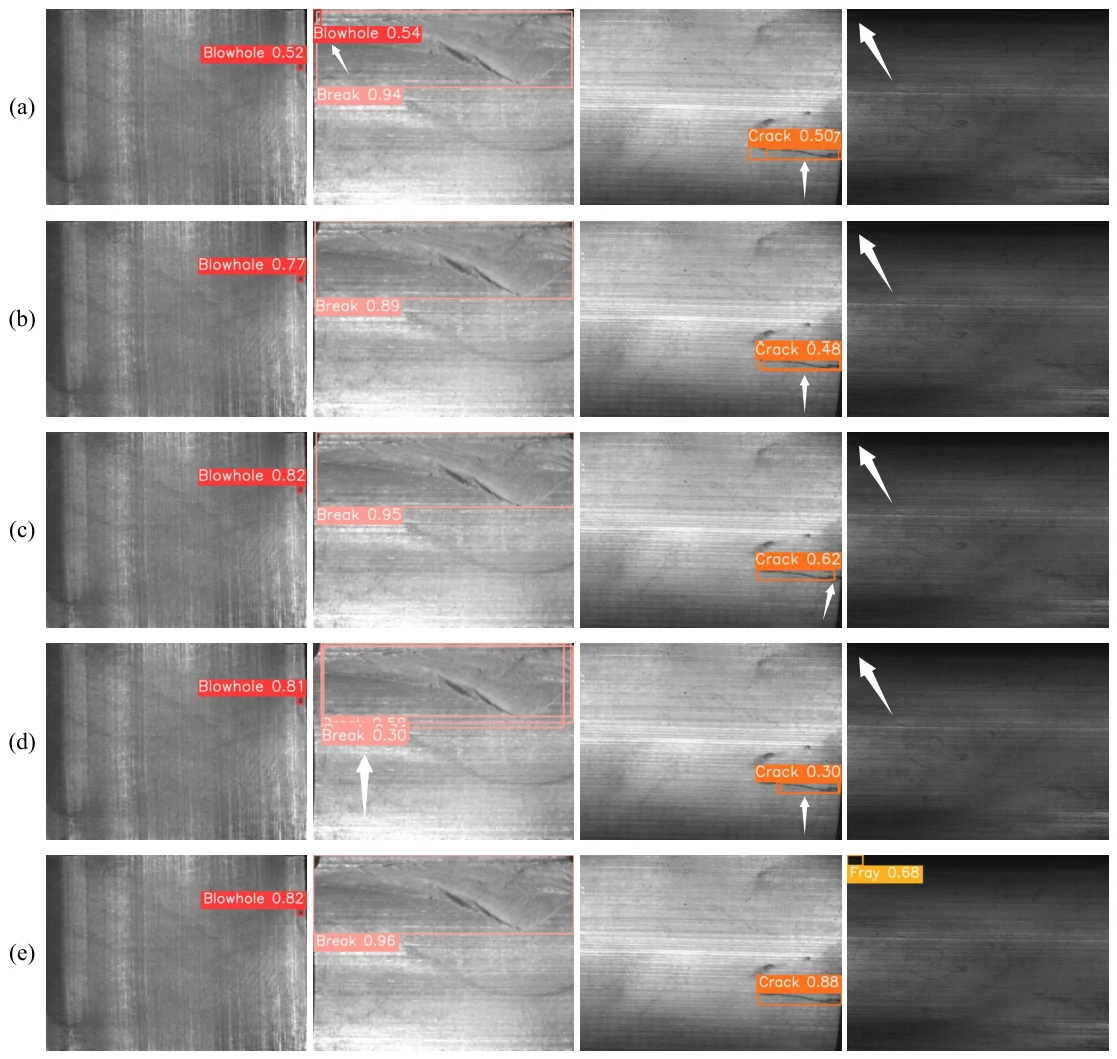
Detection results of (**a**) YOLO-RDM, (**b**) YOLO11m, (**c**) YOLO12m, (**d**) YOLO26m, and (**e**) the proposed PFG-RTDETR. White arrows indicate an additional false blowhole detection in row (**a**), column 2; multiple overlapping boxes for the same break region in row (**d**), column 2; low-confidence detections of the low-contrast crack in rows (**a**–**d**), column 3; and missed fray defects under a dark-background condition in rows (**a**–**d**), column 4.

Figure 11 further compares the detection results of YOLO-RDM, YOLO11m, YOLO12m, YOLO26m, and PFG-RTDETR, shown in rows (a)–(e), respectively. Although the compared methods identify the blowhole in the first column, their predictions differ substantially for defects affected by background interference and low contrast. In the break example, YOLO-RDM produces an additional false blowhole detection, while YOLO26m generates multiple overlapping boxes for the same break region, including a low-confidence prediction of 0.30. In contrast, PFG-RTDETR produces a single detection of the break with a confidence of 0.96, avoiding the false response and redundant boxes observed in these examples. For the low-contrast crack, YOLO-RDM, YOLO11m, YOLO12m, and YOLO26m yield detection confidences of 0.50, 0.48, 0.62, and 0.30, respectively, whereas PFG-RTDETR identifies the crack with a confidence of 0.88. The most pronounced difference appears in the fray example under a dark-background condition: all four comparison methods miss the defect, whereas PFG-RTDETR successfully detects it with a confidence of 0.68. Taken together, these examples illustrate the ability of the proposed method to detect subtle defects while avoiding background-induced false positives and redundant detections. These qualitative observations complement its higher precision, crack and fray average precision, and overall mAP reported in Table 4, supporting its effectiveness in extracting reliable defect ROIs from challenging magnetic tile images.

Overall, PFG-RTDETR achieves the highest precision, mAP_50_, and mAP_50–95_ while maintaining competitive recall. Its ability to suppress false positives in complex backgrounds, identify subtle defects, and improve localization quality provides more reliable defect ROIs for subsequent local enhancement and fine-grained segmentation.

### 4.2. Defect Enhancement Experiment Based on LACE

To further refine the quality of defect Regions of Interest (ROIs) generated by the localization model, the LACE algorithm was deployed to accentuate defect-to-background contrast while effectively suppressing interference from background noise.

#### 4.2.1. Experimental Setup and Evaluation Metrics

To ensure strict consistency and reproducibility, this experiment utilized the identical hardware and software configuration as the defect localization stage. The enhancement quality of defect ROIs was quantitatively evaluated using four representative metrics: information entropy, average gradient (AG) [52], edge intensity (EI) [53], and the patch-based contrast quality index (PCQI) [54]. For all evaluated metrics, higher quantitative values denote superior image enhancement performance.

#### 4.2.2. Results and Discussion

Quantitative evaluations of the LACE algorithm across all defect ROIs, prior to and following enhancement, are summarized in Table 5. Overall, the AG and EI metrics increased by 55.56% and 61.44%, respectively, indicating substantially heightened structural sharpness and boundary prominence across magnetic tile defects. The 9.54% gain in information entropy further confirms that LACE effectively amplifies weak defect features under severe low-contrast conditions. Specifically, LACE exhibits robust adaptability across all defect categories, maintaining PCQI values in the range of 1.17 to 1.22. For minute cracks, which are inherently challenging to segment and readily obscured by background clutter, AG and EI improved by approximately 52.4% and 63.6%, respectively, while the PCQI reached 1.210. For small blowholes with an extremely low baseline AG of 0.011, LACE elevated the metric by roughly 54.5%. Furthermore, breaks and frays demonstrated substantial gains in both AG and EI, striking an optimal balance between localized detail enhancement and global scene consistency.

**Figure 12:**
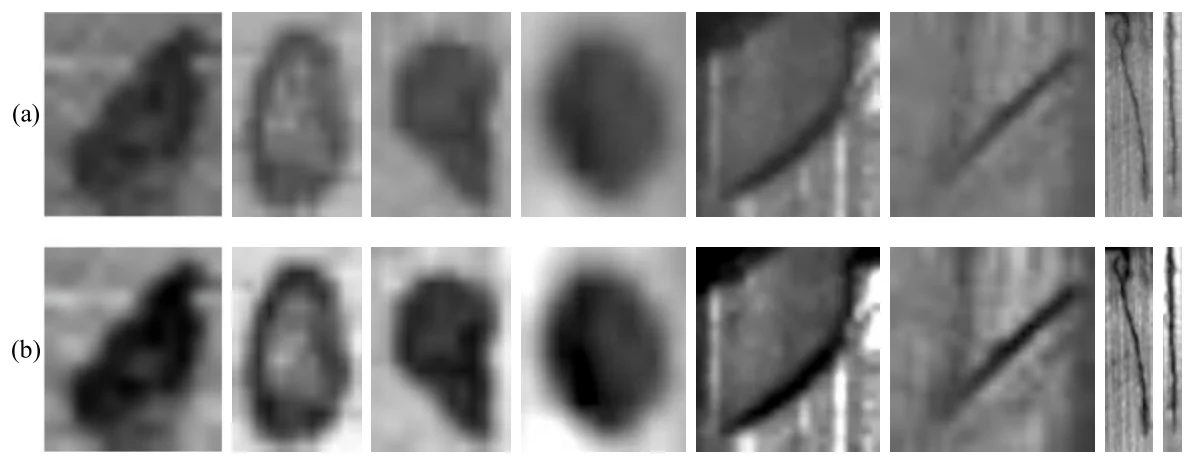
Visual comparison before and after contrast enhancement: (**a**) original image and (**b**) contrast-enhanced image.

The visualization results in Figure 12 demonstrate that LACE markedly heightens the feature saliency of magnetic tile defects. Minute cracks that are barely perceptible in the raw images are transformed into well-defined linear structures, while the boundaries of small blowholes become distinctly sharper. Furthermore, localized brightness compensation uncovers structural breaks previously obscured by surface texture. Overall, LACE not only enhances visual saliency but also expands the feature-level margin between small defects and background clutter, thereby facilitating accurate boundary delineation in subsequent image segmentation.

### 4.3. Defect Segmentation Experiment Based on CoRP

This experiment evaluates the performance of CoRP as the final segmentation module of the proposed framework. Specifically, the candidate defect ROIs generated by PFG-RTDETR are locally enhanced via the LACE algorithm and subsequently partitioned using CoRP.

#### 4.3.1. Experimental Setup

To evaluate the segmentation performance based on the output of the preceding localization and enhancement stages, this stage maintained the identical hardware and software setup to guarantee experimental reproducibility. The input dataset comprised the magnetic tile ROI images, which were localized and cropped by PFG-RTDETR and subsequently enhanced via LACE. A total of 588 enhanced ROI samples were retrieved and partitioned into CoRP training and test sets at a 9:1 ratio. In alignment with the standard CoRP protocol, all images were resized to 224×224 pixels for both training and testing, with random horizontal flipping applied to the training set for data augmentation. Following the original CoRP configuration [38], the hyperparameter for RPP iterations was set to T=3, and the PCS Top-*K* parameter was configured as K=32. Optimization was performed using the Adam optimizer with an initial learning rate of $1\times 10^{-5}$, a weight decay of $1\times 10^{-4}$, and an IoU loss for 70 epochs to ensure full model convergence.

#### 4.3.2. Evaluation Metrics

We evaluated CoRP using seven standard metrics commonly adopted in saliency detection and semantic segmentation: mean F-measure ($F_{\mathrm{mean}}$), max F-measure ($F_{\max}$) [55], structure measure (*S*-measure) [56], mean absolute error (MAE), accuracy, IoU, and the Dice coefficient. A lower MAE reflects reduced prediction error relative to the ground truth, whereas higher values across the remaining six metrics denote superior segmentation performance.

#### 4.3.3. Results and Discussion

**Figure 13:**
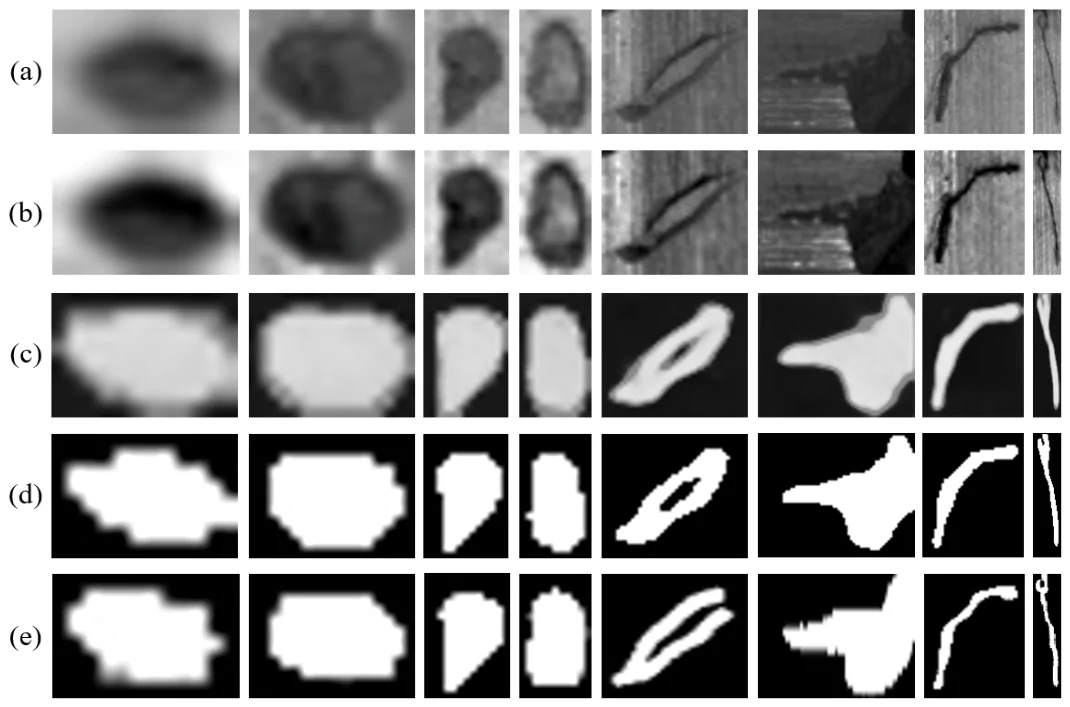
Examples of CoRP segmentation results: (**a**) original ROI, (**b**) enhanced ROI, (**c**) CoRP greyscale probability map, (**d**) binary mask obtained by Otsu thresholding, and (**e**) ground truth.

The qualitative visualization in Figure 13 demonstrates that CoRP faithfully reconstructs the morphological details of magnetic tile defects. Specifically, it extracts complete closed boundaries for small regular blowholes, preserves the topological connectivity, orientation, and endpoint fidelity of minute cracks, and accurately delineates the damaged boundaries and spatial extents of breaks and frays despite structural discontinuities or low contrast relative to background textures.

**Figure 14:**
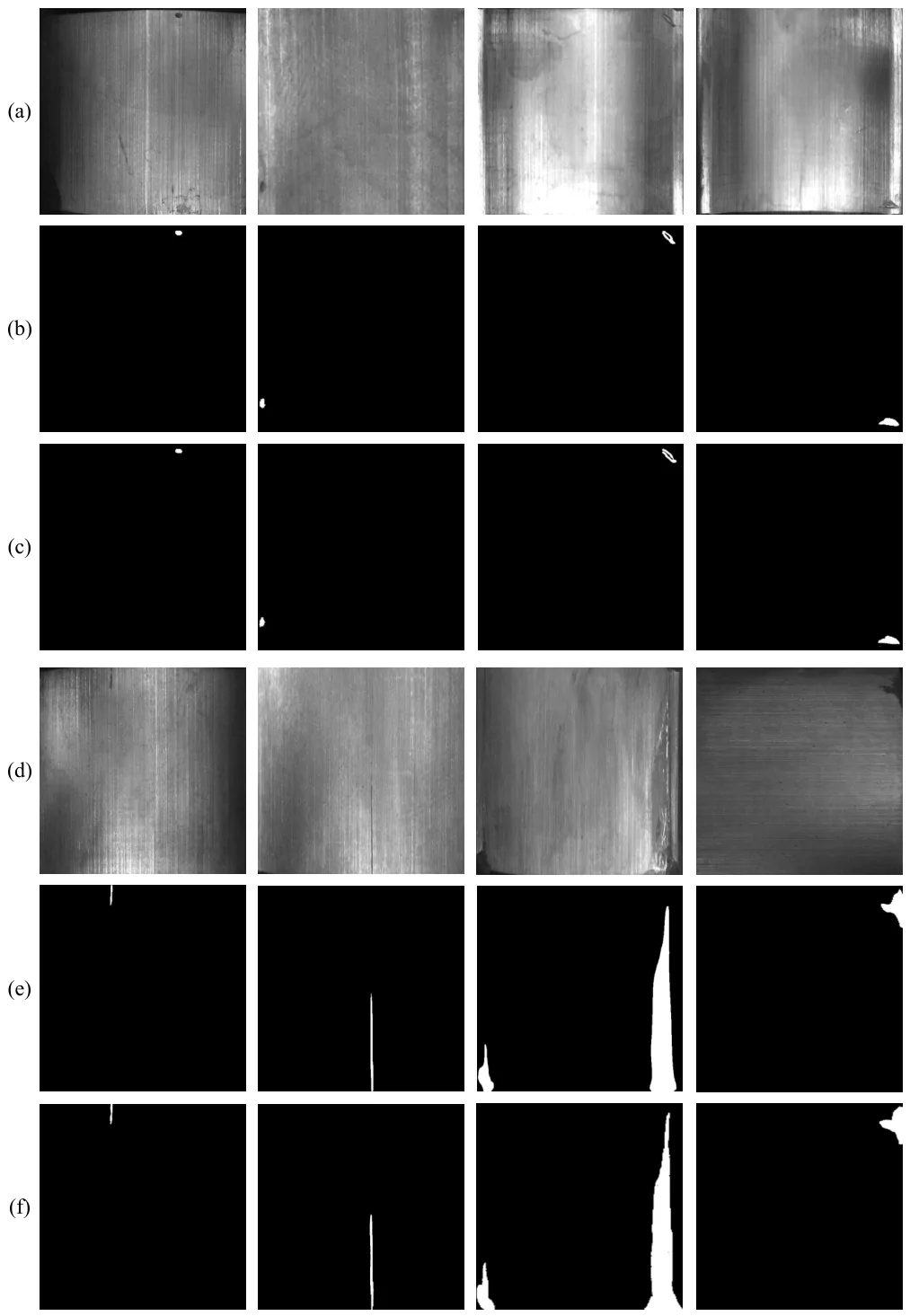
Final segmentation examples: (**a**,**d**) original images, (**b**,**e**) results of the proposed method, and (**c**,**f**) ground truth.

Finally, each binary mask was remapped to the original image dimensions for pixel-level comparison against the ground truth. As illustrated in Figure 14, the predicted boundaries demonstrate high spatial alignment with the ground truth, effectively mitigating both over-segmentation and under-segmentation.

As presented in Table 6, both configurations utilize identical PFG-RTDETR localization outputs alongside the same CoRP segmentation backend. Method 1 corresponds to direct segmentation of the raw images, whereas Method 2 performs segmentation following LACE contrast enhancement. Incorporating LACE yields consistent improvements across all evaluation metrics, with pixel accuracy rising from 99.17% to 99.34% and IoU increasing from 80.48% to 84.15%. Concurrently, precision, recall, and Dice coefficient increase by 1.84, 2.48, and 2.21 percentage points, respectively. The category-specific IoU values for blowholes, breaks, cracks, and frays reach 82.71%, 78.93%, 66.32%, and 87.13%, respectively. These quantitative findings substantiate the synergistic efficacy of LACE enhancement and CoRP segmentation in achieving high-precision magnetic tile defect inspection under low-contrast conditions.

The class-specific results also reveal differences in segmentation difficulty between frays and cracks. Despite the availability of only 32 original images, frays achieve an IoU of 87.13%. Their favorable performance may be partly attributed to spatially extended texture variations, which provide comparatively rich foreground information and contextual cues within each image. Multi-scale feature aggregation in CSP-PFGNet may help capture these irregular patterns, while ROI-based processing, LACE enhancement, and CoRP purification may further facilitate local defect discrimination and background suppression. In contrast, cracks achieve the lowest IoU among the four defect types, at 66.32%. Their slender geometry and variable widths make pixel-level segmentation particularly sensitive to small boundary errors, as even minor deviations can substantially affect the overlap of narrow foreground regions. ROI resizing and feature downsampling may weaken the representation of thin segments, while weak endpoints and irregular branches may be less consistently preserved during cross-image commonality mining and subsequent binarization. Favorable bounding-box localization therefore does not necessarily imply equally accurate crack segmentation. Incorporating LACE increases crack IoU from 63.81% to 66.32%, indicating an improvement with local contrast enhancement, while preserving fine crack structures remains a challenge.

### 4.4. Three-Stage Ablation Experiment

To validate the efficacy of the proposed localization-enhancement-segmentation framework, we conducted a comprehensive ablation study as summarized in Table 7. The six evaluated configurations comprise full-image segmentation approaches in Methods 1 and 2, alongside sequential pipelines integrating localization, ROI extraction, enhancement, and segmentation in Methods 3 to 6.

The ablation results Table 7 demonstrate that performing full-image segmentation without prior defect localization yields poor accuracy. Specifically, directly applying LACE contrast enhancement across the unsegmented full image in Method 2 causes severe performance degradation compared to the baseline full-image segmentation in Method 1. The mean F-measure collapses from 0.145 to 0.022, the max F-measure plummets from 0.340 to 0.077, and the MAE increases from 0.449 to 0.454, while the *S*-measure remains virtually stagnant at 0.403. This degradation occurs because global enhancement indiscriminately amplifies both defect signatures and vast background noise. Because defect regions occupy only a small fraction of the overall image area, the globally amplified noise dominates feature representations and suppresses defect signatures across various scales. Conversely, isolating defect ROIs via baseline RT-DETR localization in Method 3 drastically restores performance, reducing MAE to 0.229 and elevating *S*-measure to 0.773. Applying LACE inside these ROIs in Method 4 yields localized enhancement gains, reducing MAE further to 0.201. Furthermore, replacing baseline RT-DETR with the proposed PFG-RTDETR in Method 5 improves localization quality, which reduces MAE to 0.198 and increases *S*-measure to 0.790 even without enhancement. Finally, combining PFG-RTDETR localization, local LACE enhancement, and CoRP segmentation in Method 6 achieves optimal overall performance, yielding a peak max F-measure of 0.888, an *S*-measure of 0.826, and the lowest MAE of 0.170.

The ablation study systematically validates the proposed framework across its three stages along with the incremental enhancements introduced at each step. Collectively, these integrated modules effectively address the spatial sparsity of defect regions, low contrast, and severe background clutter that characterize magnetic tile surface defects.

We also evaluated the processing efficiency of the proposed framework under the same hardware environment. Table 8 reports average processing latencies of 25.49, 12.52, and 47.95 ms for PFG-RTDETR, LACE, and CoRP, respectively, corresponding to latency-derived processing rates of 39.23, 79.87, and 20.86 FPS. The full pipeline has an average latency of 86.16 ms, corresponding to 11.61 FPS. The stages have sequential data dependencies, with localization providing the ROIs for LACE and the enhanced ROIs subsequently serving as inputs to CoRP for segmentation. Among the three stages, CoRP has the highest reported processing latency under the tested conditions, making segmentation a candidate for further acceleration. These results suggest potential for online inspection, while deployment on high-speed production lines requires further optimization and evaluation against application-specific throughput and latency requirements.

### 4.5. Comparative Experiments

To benchmark the proposed framework on magnetic tile images characterized by spatially sparse defects, low contrast, and complex backgrounds, we compared it with representative segmentation and saliency-based baselines. Since the adverse effect of contrast enhancement without prior localization has been analyzed in Section 4.4, ten widely used segmentation networks were evaluated under the conventional direct segmentation paradigm, including fully convolutional network (FCN) [57], U-Net [28], PSPNet [58], DeepLabV3 [59], DeepLabV3+ [29], Semantic feature pyramid network (FPN) [60], EMANet [61], DANet [62], OCRNet [63], and CCNet [30]. In this setting, each model operates directly on full images without defect localization, ROI cropping, or localized enhancement. In addition to these deep segmentation baselines, PushU [3], a representative saliency-based method for magnetic tile surface defect detection, was also included for comparison. These baselines cover fully convolutional architectures, encoder decoder networks with skip connections, multi-scale context aggregation, attention-based long-range dependency modeling, and saliency-based defect detection. In contrast, the proposed framework follows a complete localization enhancement segmentation pipeline, in which defects are first localized, the predicted ROIs are enhanced, localized segmentation masks are generated, and the resulting masks are remapped to the original image space. For a rigorous and fair comparison, all baseline models were trained or evaluated under a unified protocol whenever applicable, using the same test set and pixel-level evaluation metrics. Specifically, the deep segmentation baselines were trained using the same configuration as CoRP, including input resolution, data augmentation, optimizer, learning rate, weight decay, loss function, and number of training epochs, without method-specific hyperparameter tuning beyond this unified setting. All methods were evaluated by pixel-level mask comparison using IoU, accuracy, precision, recall, and Dice coefficient.

As reported in Table 9, the proposed method outperforms all evaluated baselines on most metrics, achieving an IoU of 84.15%, a Dice coefficient of 91.39%, and a precision of 97.42%. Specifically, its IoU exceeds those of U-Net, DeepLabV3, and DeepLabV3+ by 3.55, 8.61, and 7.73 percentage points, respectively. Although its recall of 86.07% is slightly lower than that of U-Net (87.25%), the proposed method achieves substantially higher precision by suppressing ambiguous background responses through the CoRP mechanism. This result indicates a more conservative but cleaner segmentation behavior, which reduces false positives while maintaining competitive recall. Such a precision-oriented trade-off is particularly desirable for industrial surface inspection, where excessive false-positive regions may increase re-inspection costs and affect downstream defect size estimation. Overall, the proposed approach achieves the highest precision (97.42%) and pixel accuracy (99.34%), confirming its strong competitiveness for magnetic tile defect segmentation. Furthermore, compared with PushU, the proposed method improves accuracy, IoU, precision, and Dice coefficient by 1.65, 21.94, 32.00, and 14.69 percentage points, respectively. Although its recall is lower than that of PushU, it substantially reduces background false positives and achieves superior overall segmentation quality.

Table 10 further shows that most conventional baselines struggle to segment small-scale blowholes and fine cracks effectively. Since these defects occupy only limited spatial areas, their representations can be progressively weakened or lost during successive downsampling operations in deep convolutional architectures. For example, OCRNet and DeepLabV3+ achieve IoUs of only 17.33% and 25.16% for cracks, respectively. By contrast, the proposed method achieves IoUs of 82.71% for blowholes and 66.32% for cracks, while maintaining precision above 93% for both categories. It also obtains IoUs of 78.93% and 87.13% for breaks and frays, respectively, under challenging low-contrast conditions. Compared with PushU, the proposed method improves IoU by 70.80, 31.31, 27.67, and 12.10 percentage points for blowholes, breaks, cracks, and frays, respectively. Accuracy and precision are also improved across all four categories, demonstrating stronger defect segmentation capability and more effective background suppression. These results indicate that the proposed framework can accurately segment subtle defects while preserving the geometric morphology of larger breaks and frays.

**Figure 15:**
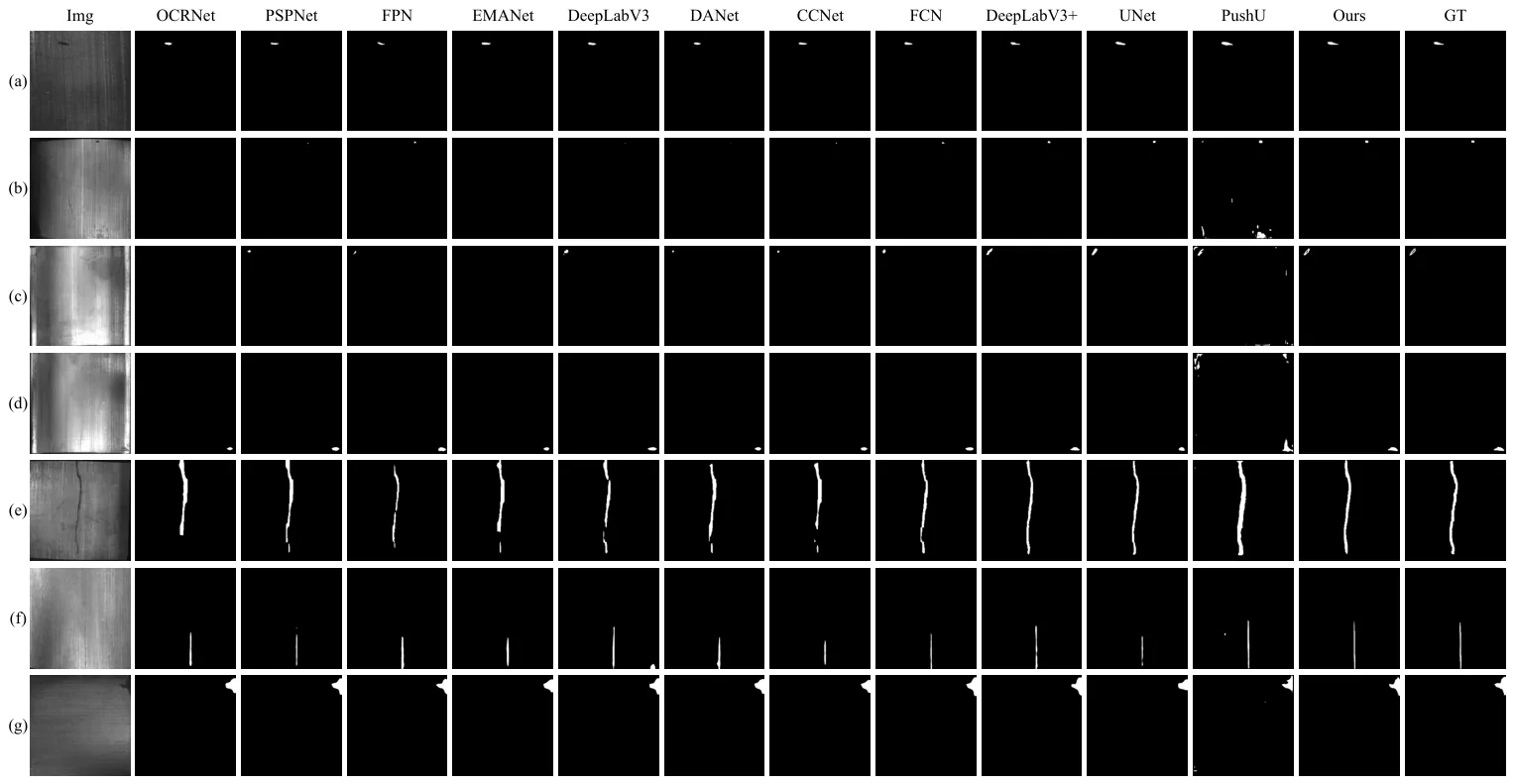
Segmentation results from different methods. (**a**) blowhole defects; (**b**) blowhole defects; (**c**) break defects; (**d**) break defects; (**e**) crack defects; (**f**) crack defects; (**g**) fray defects.

Figure 15 presents representative qualitative segmentation results from different methods. For small defects under low-contrast conditions, OCRNet, PSPNet, FPN, EMANet, DeepLabV3, DANet, and CCNet fail to extract sufficiently discriminative features in examples (b), (c), and (e), leading to missed detections or fragmented boundaries. DeepLabV3 also misclassifies background texture as false-positive defect regions in example (f). Although FCN, DeepLabV3+, and U-Net recover coarse defect topologies in example (c), they exhibit noticeable over-segmentation, and their predicted masks remain incomplete in examples (d) and (f). Although PushU detects the main defect regions, it produces pronounced spurious background responses in examples (b), (c), and (d), predicts an excessively thick crack region in example (e), and generates isolated false-positive regions in examples (f) and (g). These observations indicate that its defect coverage is accompanied by considerable interference from background textures. Across examples (a) to (g), the proposed method consistently suppresses background texture and noise interference more effectively, producing defect contours with closer spatial alignment to the ground truth.

To further evaluate the applicability of the proposed framework beyond magnetic tile inspection, additional experiments were conducted on KolektorSDD2, a public benchmark dataset for industrial product surface defect detection and segmentation [64]. The dataset comprises surface images of industrial products provided and annotated by the Kolektor Group, captured in a controlled industrial environment. It contains 3335 images, including 356 defective and 2979 defect-free samples, with image dimensions of approximately 230×630 pixels. The defects include scratches, small spots, and other surface imperfections. The proposed method was compared with FPN, DeepLabV3, U-Net, YOLO12-seg, and YOLO26-seg using accuracy, IoU, precision, recall, and Dice.

The quantitative results are reported in Table 11. The proposed method achieves the highest accuracy, IoU, recall, and Dice among the compared methods, reaching 99.49%, 74.18%, 89.21%, and 85.18%, respectively. Compared with U-Net, which obtains the second-highest IoU and Dice, the proposed method improves these two metrics by 1.08 and 0.72 percentage points, respectively, while increasing recall by 2.95 percentage points. Compared with YOLO12-seg and YOLO26-seg, it improves IoU by 4.83 and 3.97 percentage points and Dice by 3.28 and 2.68 percentage points, respectively. Although FPN achieves the highest precision of 85.79%, its recall is only 79.10%, indicating that a larger proportion of defect pixels remains undetected. In contrast, the proposed method achieves a recall of 89.21% with a precision of 81.50%, recovering more defect pixels while attaining the highest IoU and Dice. These results demonstrate improved overall segmentation performance on KolektorSDD2, with a particularly clear advantage in reducing missed defect pixels.

**Figure 16:**
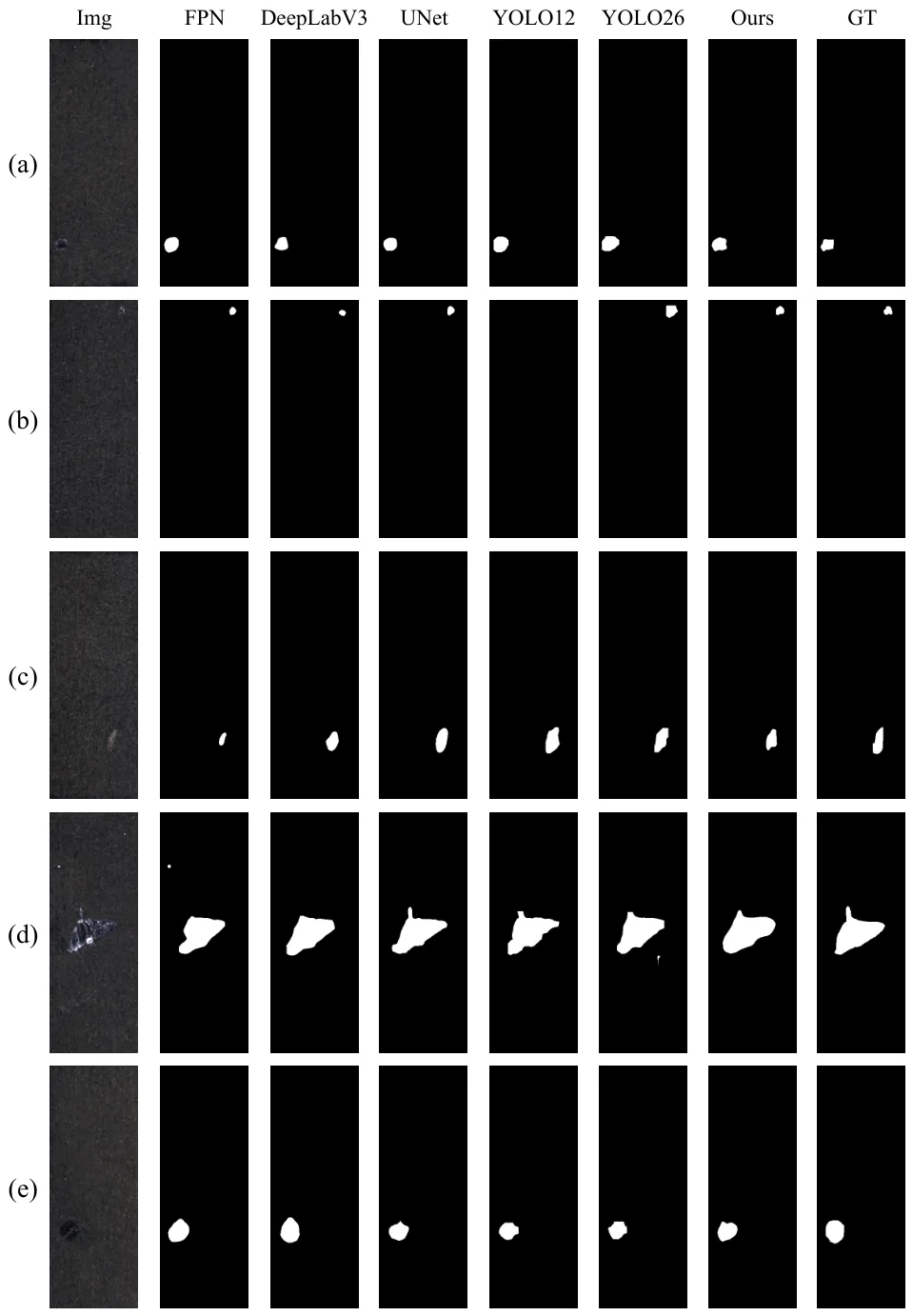
Segmentation results from different methods on the KolektorSDD2 dataset. (**a**) a lower-left defect; (**b**) an upper-right defect; (**c**) a lower-right defect; (**d**) an irregular defect; (**e**) a lower-left defect.

Figure 16 presents representative segmentation results on KolektorSDD2. The compared methods generally identify the compact defects in examples (a) and (e), but exhibit differences in the predicted extent and boundary shape. More pronounced differences are observed in the challenging examples. In example (b), YOLO12-seg completely misses the small defect near the upper image boundary, whereas the proposed method successfully recovers it. In example (c), FPN captures only a small portion of the elongated defect, while the proposed method produces a more complete segmentation of the defect region. In example (d), FPN introduces an isolated false-positive region above the main defect, and YOLO26-seg produces a small spurious response below it. The proposed method suppresses these isolated responses while retaining the main defect region. Although some differences from the ground-truth boundaries remain, these examples illustrate the ability of the proposed framework to recover small and subtle defects while limiting background interference. Together with the quantitative improvements in IoU, recall, and Dice, these observations support the applicability of the proposed framework to an additional industrial surface inspection dataset beyond magnetic tiles.

## 5. Conclusions

To address the spatial sparsity of defect regions, severe background noise, and low contrast in magnetic tile surface defect inspection, we propose a three-stage framework integrating localization, enhancement, and segmentation. Each constituent stage directly targets a distinct challenge identified in the introduction, specifically resolving weak multi-scale defect representation, background-induced feature oversmoothing, and global noise amplification during contrast enhancement.

First, in the localization stage, we developed the PFG-RTDETR model featuring a CSP-PFGNet backbone that integrates pixel-wise frequency-guided gating with large-receptive-field feature aggregation. This design suppresses low-frequency background clutter and high-frequency noise while enhancing feature extraction across multi-scale defects. Simultaneously, the AIFI-WDAM module leverages high-frequency priors to suppress background interference and mitigate the loss of subtle defect signatures caused by deep network oversmoothing. Second, in the enhancement stage, the framework applies LACE localized exclusively within defect ROIs, spatially isolating background noise and preventing the noise amplification inherent to full-image enhancement. LACE dynamically adjusts pixel values based on local statistics, sharpening gradient contrast between defects and background without introducing spurious noise. Third, in the segmentation stage, the CoRP network utilizes RPP and PCS to learn intra-class defect representations, actively filtering background interference and resolving boundary blur, missed detections, and fragmented contours commonly encountered in single-image segmentation under low contrast.

Experimental results demonstrate that the proposed framework achieves 90.91% ${\mathrm{mAP}}_{50}$ during defect localization on the magnetic tile dataset. Following localized LACE enhancement and CoRP segmentation, the system achieves an IoU of 84.15% and a precision of 97.42%. These quantitative outcomes confirm that the proposed three-stage framework effectively overcomes the core technical hurdles in magnetic tile defect inspection, exhibiting outstanding accuracy and robustness in challenging industrial environments characterized by heavy noise and subtle defect signatures. Future research will focus on generalizing this framework to diverse industrial surface inspection tasks and compressing its multi-stage architecture for real-time inference on resource-constrained edge equipment. Further efforts will focus on addressing the generalization limitations associated with the small dataset size and class imbalance and improving coordination among the three stages to mitigate error propagation within the pipeline.

## Figures and Tables

**Table 1:** Ablation experiments.

RT-DETR	CSP-PFGNet	AIFI-WDAM	mAP_50_ (%)	mAP_50–95_ (%)	Params	GFLOPs
✓	–	–	89.15	64.74	19.88 M	57.0 G
✓	✓	–	90.79	64.83	14.59 M	49.9 G
✓	–	✓	90.44	65.79	22.96 M	57.6 G
✓	✓	✓	90.91	65.68	17.68 M	50.5 G

Note: ✓ indicates inclusion of the corresponding component; – indicates its absence.

**Table 2:** Detection performance by defect type. All metric values are reported as percentages.

Model	P	R	AP50 by Defect Type				mAP_50_	mAP_50–95_
			Blowhole	Break	Crack	Fray		
RT-DETR-R18	94.35	91.75	91.90	81.57	99.50	83.64	89.15	64.74
CSP-PFGNet	91.02	92.72	93.27	82.25	99.45	88.19	90.79	64.83
AIFI-WDAM	91.51	93.49	98.26	81.63	99.50	82.35	90.44	65.79
PFG-RTDETR	93.98	92.59	96.27	81.95	99.50	85.92	90.91	65.68

**Table 3:** Localization performance across three independent training runs.

RT-DETR	CSP-PFGNet	AIFI-WDAM	mAP_50_ (%)		mAP_50–95_ (%)	
			Mean ± SD	95% CI	Mean ± SD	95% CI
✓	–	–	89.38±0.83	[87.31,91.45]	63.76±1.45	[60.17,67.35]
✓	✓	–	90.43±0.64	[88.84,92.01]	63.95±1.02	[61.42,66.47]
✓	–	✓	89.79±0.73	[87.97,91.61]	64.82±1.71	[60.56,69.07]
✓	✓	✓	90.44±0.40	[89.44,91.45]	65.06±0.61	[63.55,66.58]

**Table 4:** Performance comparison of localization methods on the magnetic tile defect dataset.

Model	P (%)	R (%)	mAP_50_ (%)				mAP_50_ (%)	mAP_50–95_ (%)	Params (M)	GFLOPs
			Blowhole	Break	Crack	Fray				
YOLOv5s+CRAB	88.61	90.24	96.72	83.20	93.68	78.58	88.05	58.33	8.34	21.96
YOLOv4+CRAB	89.25	92.24	97.43	82.40	99.24	79.50	89.65	55.98	65.41	150.5
YOLO-RDM	88.44	92.99	97.38	85.06	99.37	79.28	90.27	61.22	6.02	20.75
YOLO11m	89.57	90.60	98.13	84.59	93.80	74.22	87.69	59.89	20.06	68.20
YOLO12m	88.23	92.00	98.08	84.87	92.84	70.30	86.52	57.28	20.14	67.80
YOLO26m	87.40	85.82	97.90	88.66	95.30	69.18	87.76	56.83	21.78	74.70
PFG-RTDETR	93.98	92.59	96.27	81.95	99.50	85.92	90.91	65.68	17.68	50.5

**Table 5:** Quantitative comparison of enhancement results for magnetic tiles.

Category	AG		EI		Entropy		PCQI
	Original	Enhanced	Original	Enhanced	Original	Enhanced	
Blowhole	0.011	0.017	0.112	0.180	6.281	7.006	1.171
Break	0.022	0.032	0.185	0.293	6.353	6.957	1.196
Crack	0.021	0.032	0.184	0.301	6.294	7.019	1.210
Fray	0.023	0.037	0.163	0.269	6.484	6.858	1.214
All	0.018	0.028	0.153	0.247	6.352	6.958	1.194

Note: AG and EI denote average gradient and edge intensity, respectively. PCQI denotes patch-based contrast quality index.

**Table 6:** Quantitative results for final segmentation.

Method	Accuracy (%)					IoU (%)					P (%)	R (%)	Dice (%)
	All	Blowhole	Break	Crack	Fray	All	Blowhole	Break	Crack	Fray			
1	99.17	99.97	99.30	99.69	97.76	80.48	78.53	78.03	63.81	82.40	95.58	83.59	89.18
2	99.34	99.98	99.33	99.72	98.39	84.15	82.71	78.93	66.32	87.13	97.42	86.07	91.39

**Table 7:** Three-stage ablation experiment.

Method	RT-DETR	PFG-RTDETR	LACE	CoRP	Mean F-Measure	Max F-Measure	S-Measure	MAE
1	–	–	–	✓	0.145	0.340	0.407	0.449
2	–	–	✓	✓	0.022	0.077	0.403	0.454
3	✓	–	–	✓	0.684	0.862	0.773	0.229
4	✓	–	✓	✓	0.715	0.837	0.785	0.201
5	–	✓	–	✓	0.658	0.862	0.790	0.198
6	–	✓	✓	✓	0.676	0.888	0.826	0.170

**Table 8:** Processing latency and latency-derived processing rate of the three stages and the full pipeline.

Stage	Average Latency (ms)	FPS
PFG-RTDETR	25.49	39.23
LACE	12.52	79.87
CoRP	47.95	20.86
Full pipeline	86.16	11.61

**Table 9:** Performance comparison of segmentation methods on the magnetic tile dataset.

Model	Accuracy (%)	IoU (%)	Precision (%)	Recall (%)	Dice (%)
OCRNet	98.69	70.47	85.40	80.13	82.68
PSPNet	98.73	71.06	86.84	79.63	83.08
FPN	98.75	70.49	90.29	76.28	82.69
EMANet	98.75	72.63	83.42	84.89	84.15
DeepLabV3	98.92	75.54	86.63	85.52	86.07
DANet	98.92	73.94	92.96	78.33	85.02
CCNet	98.92	74.47	90.48	80.80	85.37
FCN	98.95	75.70	88.28	84.16	86.17
DeepLabV3+	98.99	76.42	89.16	84.25	86.64
U-Net	99.18	80.60	91.37	87.25	89.26
PushU	97.69	62.21	65.42	**92.70**	76.70
Ours	**99.34 **	**84.15**	**97.42**	86.07	**91.39**

Note: Bold values indicate the best performance for each metric among all compared methods.

**Table 10:** Class-wise performance comparison of segmentation methods on magnetic tiles.

Model	Accuracy (%)				IoU (%)				Precision (%)			
	Blowhole	Break	Crack	Fray	Blowhole	Break	Crack	Fray	Blowhole	Break	Crack	Fray
OCRNet	99.92	98.57	98.55	97.83	39.12	51.96	17.33	83.35	78.84	94.13	20.26	91.15
PSPNet	99.92	99.11	99.33	96.54	36.35	71.03	32.15	73.98	80.74	91.67	42.67	88.04
FPN	99.94	98.42	99.37	97.57	48.36	48.11	28.66	81.00	83.44	89.55	43.96	92.64
EMANet	99.92	98.73	99.29	97.22	32.03	62.09	27.84	79.65	82.55	81.03	38.92	86.39
DeepLabV3	99.93	98.91	99.20	97.74	40.60	65.28	27.94	82.99	85.05	88.41	35.83	89.41
DANet	99.92	98.71	99.60	97.69	36.87	56.52	41.06	81.78	79.35	94.49	69.00	93.47
CCNet	99.92	98.63	99.36	98.00	37.05	55.58	28.67	84.32	78.10	89.52	42.87	93.02
FCN	99.93	98.60	99.55	97.97	48.28	59.70	41.94	84.11	72.96	77.75	59.98	93.11
DeepLabV3+	99.95	99.11	98.85	98.02	64.79	70.51	25.16	84.27	83.86	93.92	28.31	94.55
U-Net	99.95	98.97	**99.74**	98.25	61.25	66.20	57.90	86.59	83.70	92.35	87.15	91.30
PushU	99.14	97.09	98.92	96.10	11.91	47.62	38.65	75.03	12.01	51.81	41.67	77.56
Ours	**99.98**	**99.33**	99.72	**98.39**	**82.71**	**78.93**	**66.32**	**87.13**	**93.87**	**97.43**	**97.53**	**97.45**

Note: Bold values indicate the best performance for each metric among all compared methods.

**Table 11:** Performance comparison of segmentation methods on the KolektorSDD2 dataset.

Model	Accuracy (%)	IoU (%)	Precision (%)	Recall (%)	Dice (%)
FPN	99.44	69.94	**85.79**	79.10	82.31
DeepLabV3	99.47	72.92	82.54	86.23	84.34
U-Net	99.48	73.10	82.73	86.26	84.46
YOLO12-seg	99.38	69.35	79.56	84.39	81.90
YOLO26-seg	99.40	70.21	80.07	85.08	82.50
Ours	**99.49**	**74.18**	81.50	**89.21**	**85.18**

Note: Bold values indicate the best performance for each metric among all compared methods.

## Data Availability

The magnetic tile dataset and the KolektorSDD2 dataset analyzed in this study are publicly available at https://github.com/abin24/Magnetic-tile-defect-datasets. (accessed on 10 October 2026) and https://www.vicos.si/resources/kolektorsdd2/ (accessed on 10 October 2026), respectively. The experimental results supporting the findings of this study are reported in the article.
